# Systematic mass-spectrometry-guided metabolic fingerprinting elucidates diversity of specialized metabolites across the Brassicaceae

**DOI:** 10.1007/s11306-026-02537-y

**Published:** 2026-09-25

**Authors:** Felicia C. Wolters, Tina Woldu, M. Eric Schranz, Marnix H. Medema, Klaas Bouwmeester, Justin J. J. van der Hooft

**Affiliations:** 1 Bioinformatics Group, Wageningen University & Research, 6708PB Wageningen, the Netherlands; 2 Biosystematics Group, Wageningen University & Research, 6708PB Wageningen, the Netherlands; 3 Department of Biochemistry, University of Johannesburg, Johannesburg, 2006 South Africa

**Keywords:** Brassicaceae, Chemotaxonomy, Computational metabolomics, *in silico* annotation, Metabolic fingerprinting, Specialized metabolites, Terpenes

## Abstract

**Background:**

Plants produce diverse bouquets of specialized metabolites (SMs), yet only a fraction of the vast phytochemical space hasbeen explored to date.Comparative analysis of SM profiles can reveal hotspots of biochemical novelty, while systematic profilingacross taxonomic levels does presently not cover large plant families.

**Materials and methods:**

To study core and accessory SM profiles in the Brassicaceae plant family, we fingerprinted 14 species by Liquid-ChromatographyMass-Spectrometry (LC-MS/MS). We develop standardized experimental and computational workflows integrating in silicoannotation tools to study consensus compound class and substructure distributions of SMs. Furthermore, we investigate thecongruence of chemotaxonomy and species phylogeny across an extended panel of 17 species.

**Results:**

Unique metabolite profiles were outstanding in *Camelina sativa, Capsella rubella, and Barbarea vulgaris*, with the largest uniqueterpenoid profile annotated in C. sativa, accounting for 33.5% and 55.6% in positive and negative ionization mode, respectively.Substructure motifs were found to overlap with compound class predictions, highlighted for triterpenoids in Camelinodae. Furthermore, dual-tissue chemotaxonomic clustering resembled relationships of *Brassica nigra*, B. *oleracea*, B. *carinata*, and B. *juncea*, and corresponding B and C subgenomes across tissues.

**Conclusion:**

We anticipate that our systematic approach can serve as a blueprint for investigating biochemical diversity in other plant lineagesand can boost the characterization of plant natural product pathways.

**Supplementary Information:**

The online version contains supplementary material available at https://doi.org/10.1007/s11306-026-02537-y.

## Significance

Plants produce immense chemical diversity, yet biochemical divergence among species is poorly understood. Using standardizedmetabolomics and computational annotation, we characterize core and accessory metabolite profiles across taxonomic levels in theBrassicaceae and identify major hotspots of biochemical novelty at a family-wide scale.

**Supplementary Information:**

The online version contains supplementary material available at https://doi.org/10.1007/s11306-026-02537-y.

## Introduction

Plants rely on a vast array of chemical defenses to compensate for their sessile nature, representing one of the most structurally diverse and largely unexplored metabolite suites. Throughout human history, the rich chemical space of plant specialized metabolites (SMs) has been explored for its potential applications in pharmacy and food industry, with numerous examples of plant-derived drugs (Wang et al., 2019), food additives (Costa-Pérez et al., 2023), and applications for crop resistance breeding (Afifah et al., 2019; Macel et al., 2019; Tinte et al., 2021). Our understanding of chemodiversity in plants has grown in scale with the aid of novel computational tools, utilizing the increasing amount of high-resolution data from Gas and Liquid Chromatography tandem Mass Spectrometry (GC- and LC-MS/MS) platforms. Depending on the methods used for compound extractions (e.g. choice of solvents, gradients, instrument types), metabolic profiling captures structural diversity under positive and negative ionization modes at high resolution. In recent years, metabolic fingerprinting studies explored the metabolite diversity across multiple taxonomic levels, including plant family (Kang et al., 2019; Liu et al., 2021), genus (Elser et al., 2023) and species (Bakir et al., 2020; Fiehn et al., 2000; Houshyani et al., 2012; Padilla-González et al., 2023; Qiu et al., 2024) on an extensive scale. Intraspecific SM profiles were captured across genotypes (Tohge et al., 2018), individual plants (Mönchgesang et al., 2016) and tissues, including leaf and root tissue (Marzouk et al., 2023), seeds and fruits (Anaia et al., 2025; Barreda et al., 2024, 2025; Farag et al., 2024; Nguyen et al., 2025).

Plant adaptation to ecological niches is further differentiated by ontogeny and biotic and abiotic factors, with specialized biochemistry co-evolving with herbivores, pathogens, and microbiomes (Anaia et al., 2025; Goodger et al., 2013; McLaughlin et al., 2023). Yet, research focusing on metabolic fingerprinting does often not incorporate dimensions of tissues across species taxonomy within the same study. Consequently, the systematic study of chemical diversity across plant evolutionary history is still in its infancy, partially due to limitations in reliable compound annotation and integration of LC-MS/MS data across platforms, experiments, and batches (Alseekh & Fernie, 2023; Houriet et al., 2025).

In recent years, the options for large-scale and pan-repository metabolomics data integration have been facilitated by the advancement of computational strategies. Nevertheless, metabolomics data integration across batches and experiments is still hampered by shifts in retention time (RT) and differences in instrument settings, interfering with the alignment based on RT windows and fragmentation patterns (Hajjar et al., 2023). Beyond sensitivity to technical settings in LC-MS/MS analysis, metabolic fingerprints are determined by biological variation during plant development, and by environmental conditions, underscoring the need for standardization of experimental setups. Hence, the systematic collection of LC-MS/MS data under standardized conditions is a prerequisite for robust comparative analysis, and identification of chemodiversity hotspots (Wolters et al., 2024). Contextualization of comparative SM profile analysis across plant taxonomy further relies on the resolution of species phylogeny. Plant families such as the Brassicaceae (crucifers) have been studied extensively to model evolutionary trajectories of genomic rearrangements after polyploidization (German et al., 2023; Hendriks et al., 2023; Walden & Schranz, 2023).

The Brassicaceae family is known for its economically important species cultivated as vegetable and oil crops, ornamentals, and the model species *Arabidopsis thaliana*. Recently, a comprehensive phylogeny of the Brassicaceae family has been released. Together with a high-resolution phylogeny, Hendriks et al. (2023 ) introduced a revised family classification, covering two subfamilies (Brassicoideae and Aethionemoideae) and five supertribes (Camelinodae, Brassicodae, Arabodae, Heliophilodae, Hesperodae). The evolution of glucosinolates in the Brassicaceae family as a characteristic SM class has been studied extensively (Agerbirk et al., 2021; Bird et al., 2025). Other compound classes such as triterpenoid saponins were exclusively found in one genus (*Barbarea*) in the Brassicaceae (Erthmann et al., 2018; Li et al., 2023). Yet, untargeted metabolic fingerprinting studies do presently not cover multiple related species across supertribes in the Brassicaceae family.

Here, we present a systematic comparative analysis of SM class distributions across the Brassicaceae, focusing on the supertribes Camelinodae and Brassicodae. To investigate shared and unique SM profiles, we collected and analyzed LC-MS/MS data from 14 Brassicaceae species and two tissues (leaf and root), incorporating feature extraction in mzmine, computational annotation of extracted metabolite features, and feature-based molecular networking (FBMN) in both positive and negative ionization mode. We specifically highlight the shared metabolic profile - hereafter referred to as “core-metabolome” - across supertribes, genera and species, and in both leaf and root tissue. In addition, we investigate the distribution of alkaloid and terpenoid SM classes across taxonomic levels and tissues, and examine the distribution of substructures corresponding to oleanane triterpenoid compound class annotations. Ultimately, we compared chemotaxonomic relationships based on hierarchical clustering with species phylogeny across an extended set of 17 species, to determine congruency across tissue-specific metabolite profiles.

**Fig. 1:**
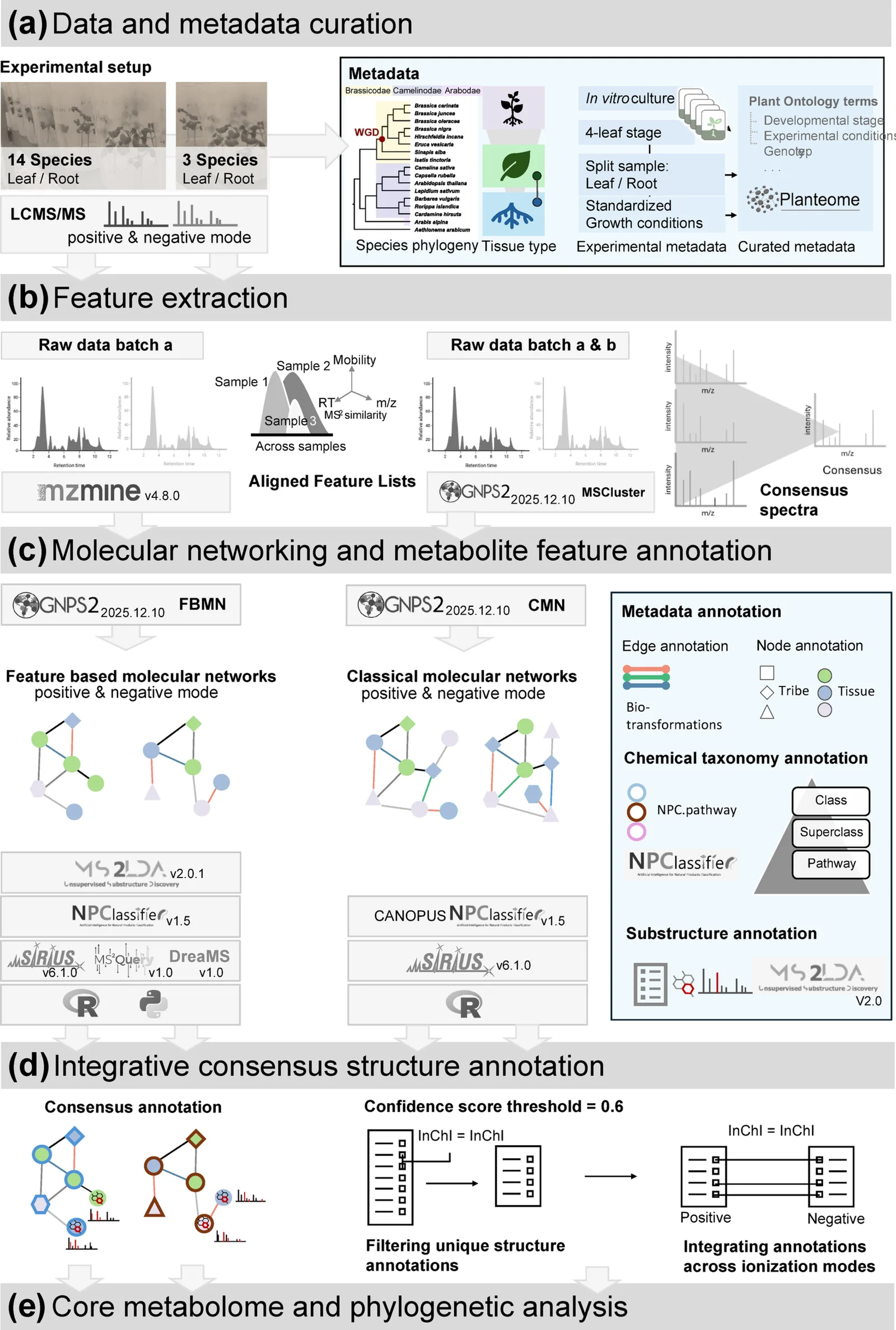
Workflow for untargeted metabolomics data collection, metadata curation, compound annotation, and batch integration for chemical taxonomy analysis. **a** Data and metadata curation. Batches were analyzed independently on the LC-MS/MS platform using the same protocol. **b** Feature extraction was conducted in mzmine for batch “a” including 14 species. Converted MZXML files of batch “a” and “b” including in total 17 species were submitted to the GNPS2 online platform, utilizing MSCluster for clustering spectra based on cosine similarity. **c** Molecular networking and metabolite feature annotation. Batch “a” and combined batches “a” and “b” were submitted for Feature-Based Molecular Networking (FBMN) and Classical Molecular Networking (CMN) to the GNPS2 platform, respectively. The FBMN was annotated using SIRIUS including CANOPUS, MS2Query, and DreaMS, and chemical ontology annotations were obtained from NP.Classifier. Batch “b” was annotated using SIRIUS with a cutoff confidence exact score of 0.6. **d** Integrative and consensus structure annotations were obtained by matching structural and chemical ontology predictions separately for annotation tools in batch “a”. In batch “b”, similar structure predictions were merged across multiple features, and across ionization modes. **e** Core metabolome and phylogenetic analysis were conducted for the datasets including 14 and 17 species, respectively

## Results and discussion

### The “core-metabolome” reflects taxonomic relationships in the Brassicaceae

To study the composition of conserved metabolic fingerprints in the Brassicaceae family, mass features extracted from LC-MS/MS data were aligned across 14 species in the Camelinodae and Brassicodae supertribes, and subsequently annotated with chemical class terms according to the NP.Classifier chemical ontology (Kim et al., 2021). Mass features represent detectable ions of compounds in a complex mixture. In total, 7,683 and 6,433 mass features from raw LC-MS/MS spectra were extracted and aligned with mzmine in positive and negative ionization mode, respectively (Fig. 1). Clustering of quality control (QC) samples was assessed using principal component analysis (PCA), and overlap of detected features between QC samples and plant tissue extracts was evaluated in positive and negative ionization modes in the accompanying data descriptor study (Wolters et al., 2026).

**Fig. 2:**
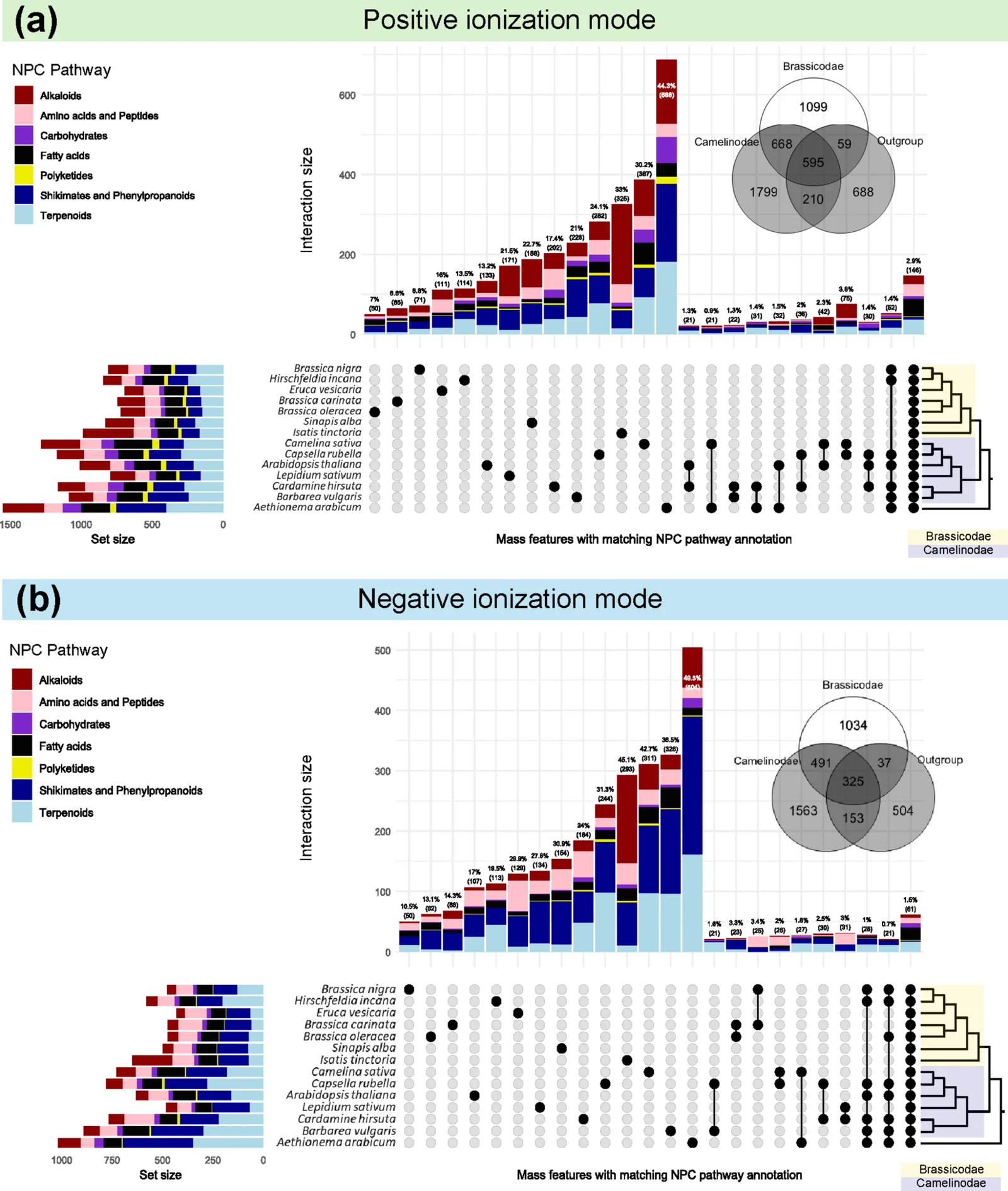
Distribution of mass features across 14 Brassicaceae species under positive and negative ionization mode. Untargeted metabolomics (LC-MS/MS) data was filtered for consistent annotations using computational annotation tools (SIRIUS, MS2Query, DreaMS) on the NP.Classifier pathway chemical ontology level. Venn diagrams show overlaps of aligned features across supertribes. Only sets with at least 20 metabolite features are shown. **a** Metabolite features annotated in positive ionization mode. **b** Metabolite features annotated in negative ionization mode

Filtering for consistent NP.Classifier pathway annotations across annotation tools (SIRIUS, DreaMS against the MassSpecGym library, MS2Query) resulted in 5,118 features in positive ionization mode (Fig. 2a), and 2,542 features in negative ionization mode (Fig. 2b). The overlap among the three computational annotation tools, as well as the distribution of partial (two out of three tools) and full (three out of three tools) agreement in structural and compound class annotations was assessed separately for the predicted compound classes. Further details are provided in Wolters et al. (2026). Notably, the applied analytical method (reverse-phase LC-MS/MS) captures only a subset of plant-produced metabolites, primarily semi-polar to moderately polar compounds within a particular mass range that are extractable with the selected solvent. Consequently, the compounds presented here represent only a portion of the plant’s chemical space measured under standardized controlled conditions for comparative analysis. Therefore, the term “core metabolome” refers to the subset of the plant chemical space that is accessible using the applied methods.

The “core” set of annotated metabolites in the Brassicaceae shared by at least one species within Camelinodae, Brassicodae, and the outgroup is represented by 595 (11.6%) metabolite features in positive ionization mode, and 325 (12.8%) features in negative ionization mode (Fig. 2a). Brassicodae and Camelinodae share 668 (13%) metabolite features in positive ionization mode (Fig. 2a), and 491 (19.2%) features in negative ionization mode (Fig. 2b), representing the “core metabolome” shared by both supertribes. The unique metabolite profile of the outgroup species *Aethionema arabicum* is comparable in size with the unique profiles of both supertribes, with 13% in positive and 19.8% in negative ionization mode, suggesting balanced representation of both supertribes and the outgroup. A smaller fraction of annotated metabolite features is conserved on the species level, with 2.9% (146) and 1.5% (61) of all consistently annotated features shared across all species in positive and negative ionization mode, respectively.

Our findings suggest that conserved profiles across supertribes reflect phylogenetic distance, highlighted by the overlaps of Camelinodae, Brassicodae and outgroup in both ionization modes. However, unique metabolite features represent a broad range of 7% to 45% of the total captured fingerprint, especially pronounced in the Camelinodae and under negative ionization mode. The higher numbers of species-specific detected and consistently annotated metabolite features in Camelinodae compared with Brassicodae can only partly be attributed to the larger overall feature set observed in Camelinodae species. Notably, species representing the Camelinodae supertribe also cover larger phylogenetic distances compared to Brassicodae species. The represented tribes within the Camelinodae supertribe cover Isatidae, Camelinae I, Arabidopsidae, Lepidae, and Cardaminae, while the Brassicodae supertribe is mainly limited to species sampled from the Brassiceae tribe. Recent meta-analyses indicate that, although the discovery of new plant compounds shows signs of saturation as more species are studied, the known chemical space still represents only a small fraction of plant diversity (Domingo-Fernández et al., 2023; Hart et al., 2025). To better estimate undiscovered chemical diversity and reveal shared biochemical space, we propose systematic metabolomic fingerprinting across more taxonomic levels within the Camelinodae supertribe.

**Fig. 3:**
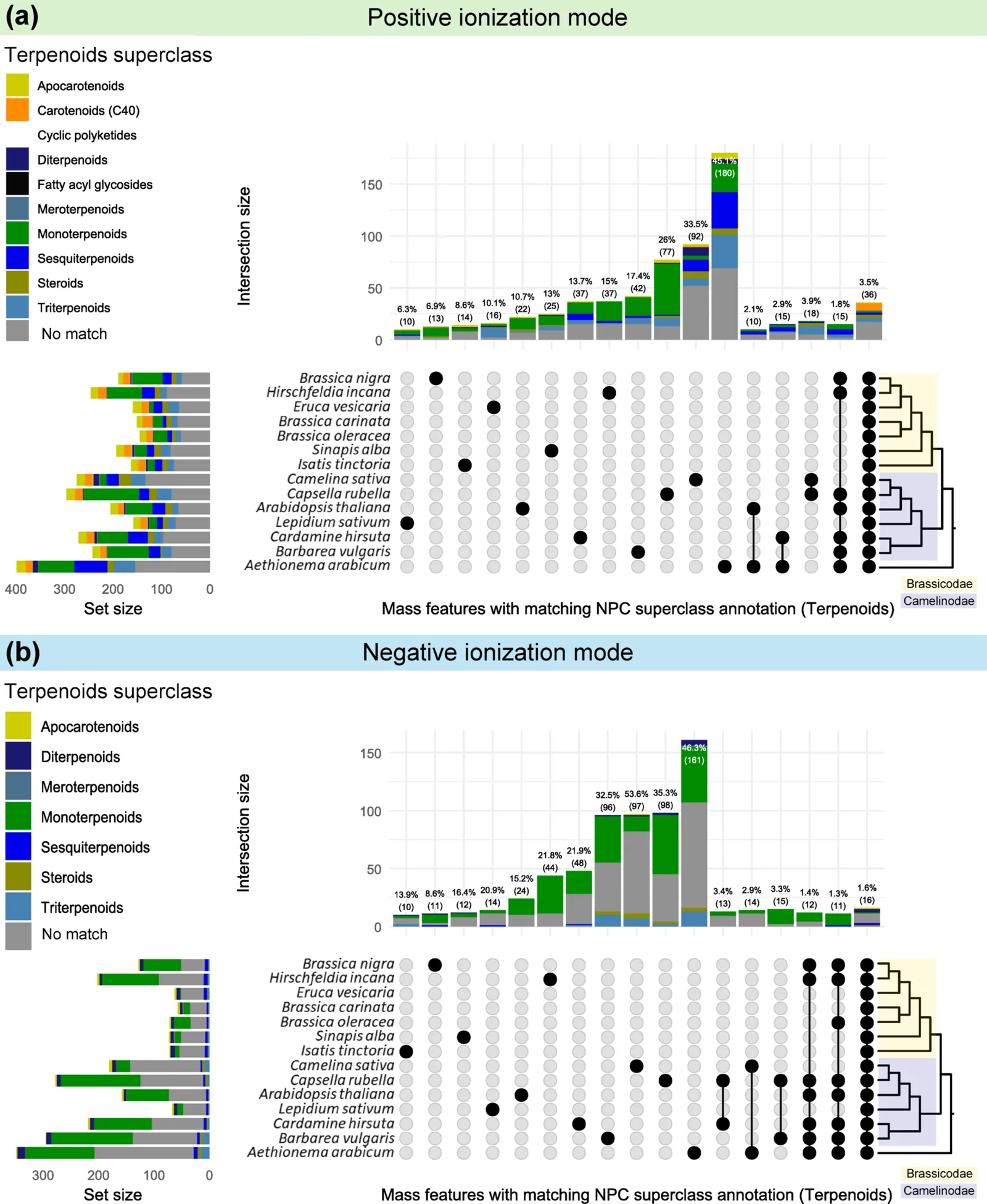
Distribution of mass features across 14 Brassicaceae species under positive and negative ionization mode. Untargeted metabolomics (LC-MS/MS) data was filtered for consistent annotations using computational annotation tools (SIRIUS, MS2Query, DreaMS) on the NP.Classifier superclass chemical ontology level, and for “terpenoid” pathway annotations. Terpenoid pathway annotations with inconsistent superclass annotation across at least two annotation tools are indicated in grey (“no match”). Venn diagrams show overlaps of aligned features across supertribes. **a** Metabolite features annotated in positive ionization mode. **b** Metabolite features annotated in negative ionization mode

### Substructures associated with oleanane triterpenoids and conjugated glycosides are not exclusive to *Barbarea vulgaris*

**Fig. 4:**
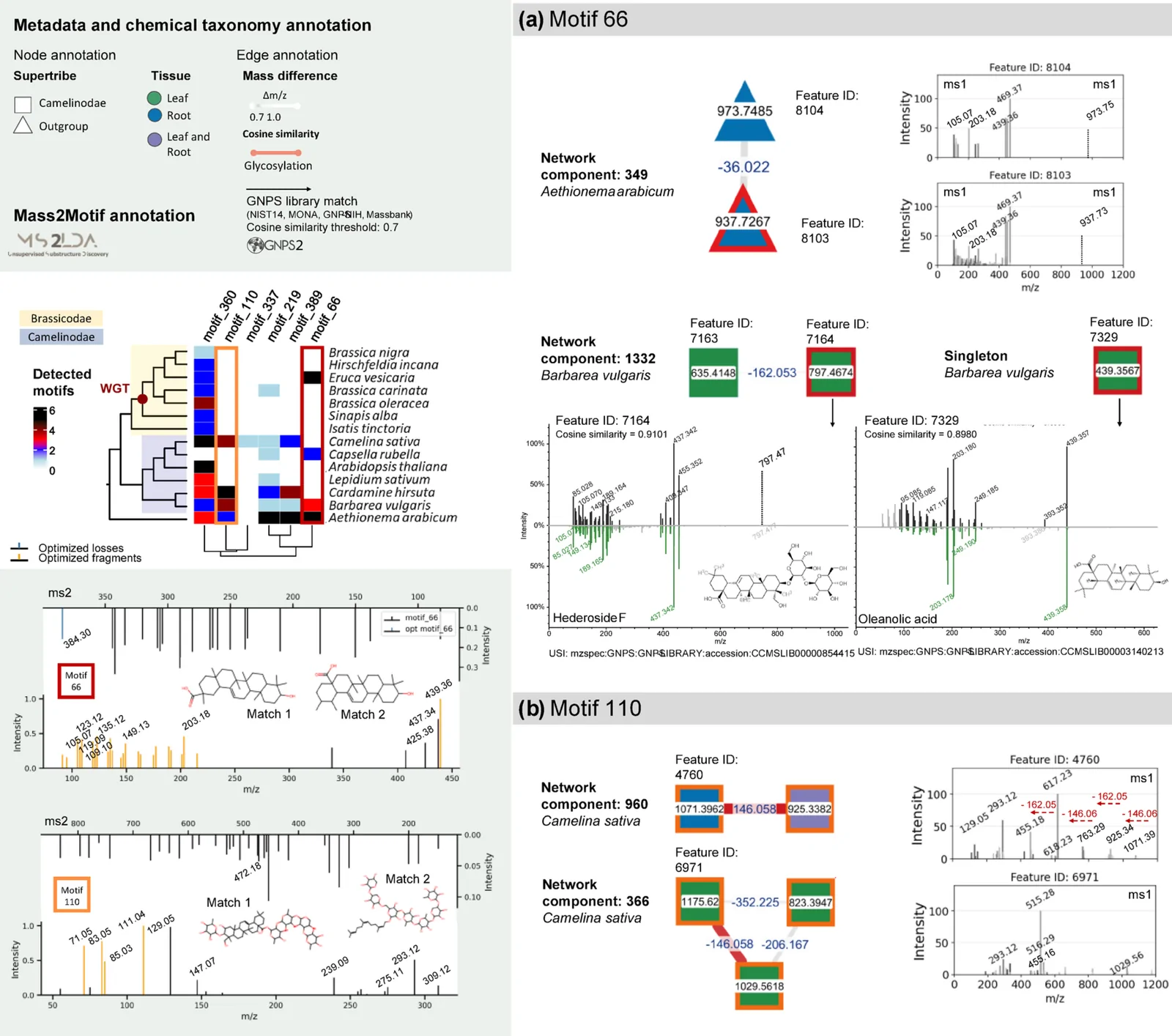
Mappings of Mass2Motifs associated with putative triterpenoid saponins extracted with MS2LDA in positive ionization mode. Mass2Motifs associated with fragmentation patterns and annotated substructures of oleanane-type triterpenoids were mapped on molecular families in the Feature-Based Molecular Network (FBMN). Node shape and color represent presence/absence in supertribes and tissues, respectively. Edges were annotated with biotransformations corresponding to glycosylation (162.05 Da, 146.06 Da) and hydroxylation (18.01 Da). **a** Distribution of motif_66 and motif_219 across features with matching triterpenoid superclass prediction according to NP.Classifier chemical ontology, and library matches for spectra found in *Barbarea vulgaris*. **b** Distribution of motif_110, and corresponding spectra of molecular features in *Camelina sativa*

Presence of consensus class annotations in Camelinodae species indicated distribution of putative triterpenoid metabolite features in species belonging to the Camelinodae supertribe Fig. 3). In *B. vulgaris*, compound class predictions overlap with library matches obtained for previously described oleanane triterpenoid saponins (Khakimov et al., 2016; Shinoda et al., 2002; Fig. 4). To our knowledge, triterpenoid saponins have not been described previously in other species of the Brassicaceae family apart from *B. vulgaris*. To investigate the distribution of putative triterpenoid saponin-like metabolite features, molecular families including consistent annotations were subjected to detailed analysis. To investigate the overlap of oleanane triterpenoid annotations with shared substructures, patterns of mass fragmentation and neutral losses (Mass2Motifs) were extracted using MS2LDA in both positive (Fig. 4) and negative ionization mode (Fig. S6). Extracted Mass2Motifs were inspected with regards to substructure SMILES annotations and characteristic MS fragmentation patterns corresponding to oleanolic acid (Wikidata: Q418628) and conjugated triterpenoid saponin glycosides reported in the literature (Khakimov et al., 2016; Shinoda et al., 2002).

Three Mass2Motifs detected in positive ionization mode spectra were annotated as substructures of putative oleanane- and ursane-type triterpenoids. Motif_66 and motif_219 (Fig. S6) were found to be associated with an oleanolic acid substructure, whereas motif_110 matches conjugated oleanane triterpenoid glycosides (Fig. 4b). These motifs were detected across leaf and root tissue of five Camelinodae species, in *Brassica carinata* (Brassicodae), and in the outgroup *A. arabicum*. Pseudospectra of motif_66 and motif_219 resembled characteristic fragment peaks of oleanane-type triterpenoids (*m/z* 439.36, 119.09, 105.07) which were also found in library spectra of oleanolic acid (GNPS-Library accession: CCMSLIB00003140213) and referenced in the literature (Fig. 4a). Base peaks in the pseudospectrum of motif_66 at *m/z* 439, 437, 425 corresponded to triterpenoid saponin aglycones previously reported by Trugawa et al. (2019). Also, the distribution of motif_66 in metabolite features was found aligned with the triterpenoid superclass annotations from NP.Classifier, and with GNPS library matches for oleanolic acid and hederoside F (Wikidata: Q105301871) in *B. vulgaris*, which also share a consensus class annotation as putative oleanane-type triterpenoids. Another characteristic base peak (*m/z* 471) of oleanane-type triterpenoid saponins in *B. vulgaris* was detected in the MS1 spectra of *A. arabicum*. This peak occurred in two mass features with an analog match to betulonic acid methyl ester (Wikidata: Q105337617) predicted by SIRIUS and MS2Query, and associated with motif_66 below the probability score threshold (probability score of 0.45; Material S6).

Motif_66 was extracted from spectra of 16 metabolite features (probability score > 0.5, overlap score > 0.15) in positive ionization mode. Of these, 14 features were consistently annotated as triterpenoids by NP.Classifier. Furthermore, motif_66 was found to overlap with GNPS library matches for previously described triterpenoid saponins in *B. vulgaris*. For two features annotated with motif_66 in *B. vulgaris*, spectral matches to oleanolic acid and hederoside F were obtained in the GNPS library. Notably, the aglycone peaks (*m/z* 437, 455) in the library spectrum of hederoside F overlap with motif_66 (*m/z* 437) and motif_219 (*m/z* 455), and are also shared by features annotated with motif_110 in *C. sativa* (Fig. 4a, Material S3). In addition, MS1 spectra of features sharing motif_110 exhibit characteristic putative aglycone peaks at *m/z* 445 and 455 that were not part of the MS2 motif. Another two features in *C. sativa* share hexoside (162.05 Da) and rhamnoside (146.06 Da) sugar losses in MS1 spectra (Fig. 4b). Optimized fragment peaks in motif_110 (*m/z* 71.05, 83.05, 85.03, 111.04) do not overlap with motif_66 and motif_219, but were annotated as fragments indicative of a glycosylation according to MotifDB (reference MotifSet: Massbank derived Mass2Motifs). Notably, molecular families annotated with motif_110 contained metabolite features with highly similar retention time, indicating in-source fragmentation (ISF). The impact of ISF on metabolite feature annotation in untargeted LC-MS/MS has been discussed recently (El Abiead et al., 2025). Therefore, putatively identified glycosylation patterns needed to be examined carefully. Nonetheless, metabolite features prioritized by overlapping compound class and substructure annotations display characteristic fragmentation patterns of triterpenoid saponins in positive (Fig. 4) and negative ionization mode (Fig. S6) that have not been reported in *C. sativa*,* C. hirsuta*,* C. rubella* and *A. arabicum* before.

### Consensus annotations of structures and sub-structures align with library matches of putative tryptophan alkaloids, lignan glycosides, and monoterpenoids

**Fig. 5:**
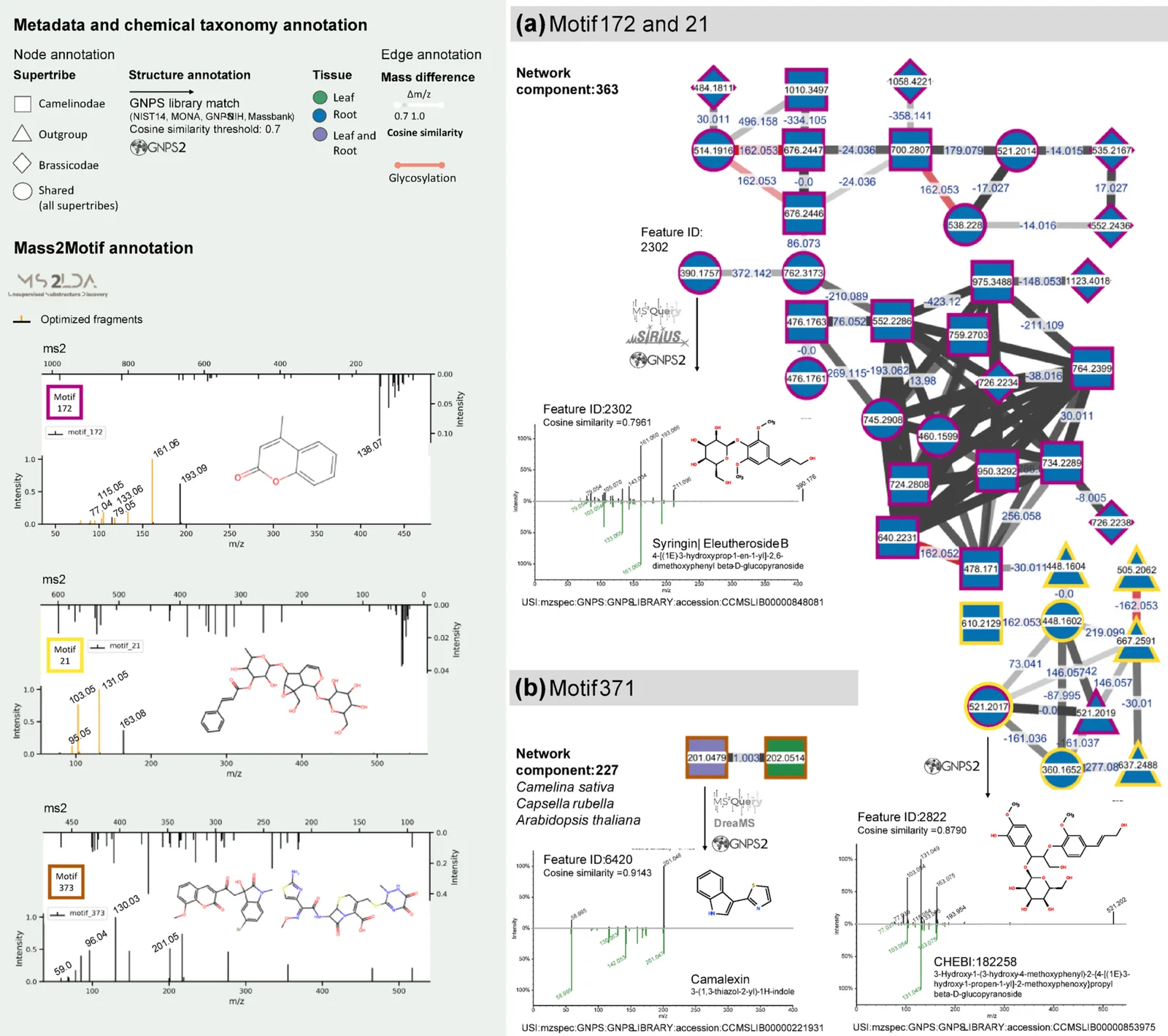
Mappings of Mass2Motifs associated with putative tryptophan alkaloids, lignan glycosides, and monoterpenoids extracted with MS2LDA in positive ionization mode. Mass2Motifs associated with fragmentation patterns and annotated substructures were mapped on molecular families in the Feature-Based Molecular Network (FBMN). Node shape and color represent presence/absence in supertribes and tissues, respectively. Edges were annotated with biotransformations corresponding to glycosylation (162.05 Da). **a** Distribution of motif_172 and motif_21 across features with matching triterpenoid superclass prediction according to NP.Classifier chemical ontology, and library matches for spectra found in all supertribes. **b** Distribution of motif_371, and corresponding spectra of molecular features in *Camelina sativa*, *Capsella rubella* and *Arabidopsis thaliana*

Consensus annotations on pathway and superclass levels revealed the distribution of metabolite features from multiple chemical classes across all supertribes. In positive and negative ionization mode, alkaloid profiles were particularly enriched in tryptophan alkaloid superclass annotations across all species. Anthracillic acid alkaloids were specifically abundant in I. *tinctoria*, whereas ornithine alkaloids were most abundant in *A. arabicum* in positive ionization mode (Fig. S3). To investigate the alignment of computational structure and sub-structure annotations, library matches and molecular family composition, we examined two molecular families containing metabolite features with library matches to syringin (GNPS compound ID: NCGC00180873-02, Wikidata: Q105222928), camalexin (Wikidata: Q19903715), and an additional lignan glycoside (GNPS compound ID: NCGC00381098-01). Detected Mass2Motifs were subsequently mapped to assess the correspondence between computational annotations (Fig. 5). Syringin and camalexin have been extracted earlier from various members of the Brassicaceae (Lopa et al., 2025; Ke et al., 2026). The phytoalexin camalexin has been specifically extracted from *A. thaliana and C. sativa* in earlier research (Lopa et al., 2025). In addition, our analysis indicated the presence of camalexin in root tissue of *C. rubella* (Fig. 5b). Motif_172 and motif_21 were widely distributed in network component 363, pointing to the presence of indole ring substructures observed in library matches and corresponding consensus structure annotations (Fig. 5a). The MotifDB annotation of motif_373 indicated alkylamine substructures (nitrogen-containing substructures, score: 0.1572, aligning with the presence of fragments observed in the corresponding spectrum of camalexin (Fig. 5b). Our analysis suggested that computational consensus annotations of compound classes, structures and sub-structures yielded complementary information on characteristic properties of metabolite features, aiding in the propagation of annotations through the topology of the molecular network. To further elucidate the specificity of camalexin within the Camelinodae supertribe, we suggest systematically fingerprinting closely related species within the tribes Arabidopsidae and Camelinae I.

### Chemical taxonomy across 17 species aligns largely with species phylogeny when combining leaf and root tissue profiles

The diversification of plant specialized metabolites has long been linked to the theory of the coevolutionary arms race in plants, which proposes that plant chemical diversity is shaped by reciprocal interactions with biotic factors, thereby influencing evolutionary relationships among plant species (Pichersky & Raguso, 2018). However, tissue-specific incongruences between metabolic and phylogenetic distance suggest that different plant organs may be subject to distinct selective evolutionary pressure (Anaia et al.^2025^; Nguyen et al., 2025). To investigate inconsistent degrees of conservation in metabolic profiles of leaf and root tissue across related plant species, hierarchical clustering of tissue profiles was independently compared to phylogenetic relationships.

**Fig. 6:**
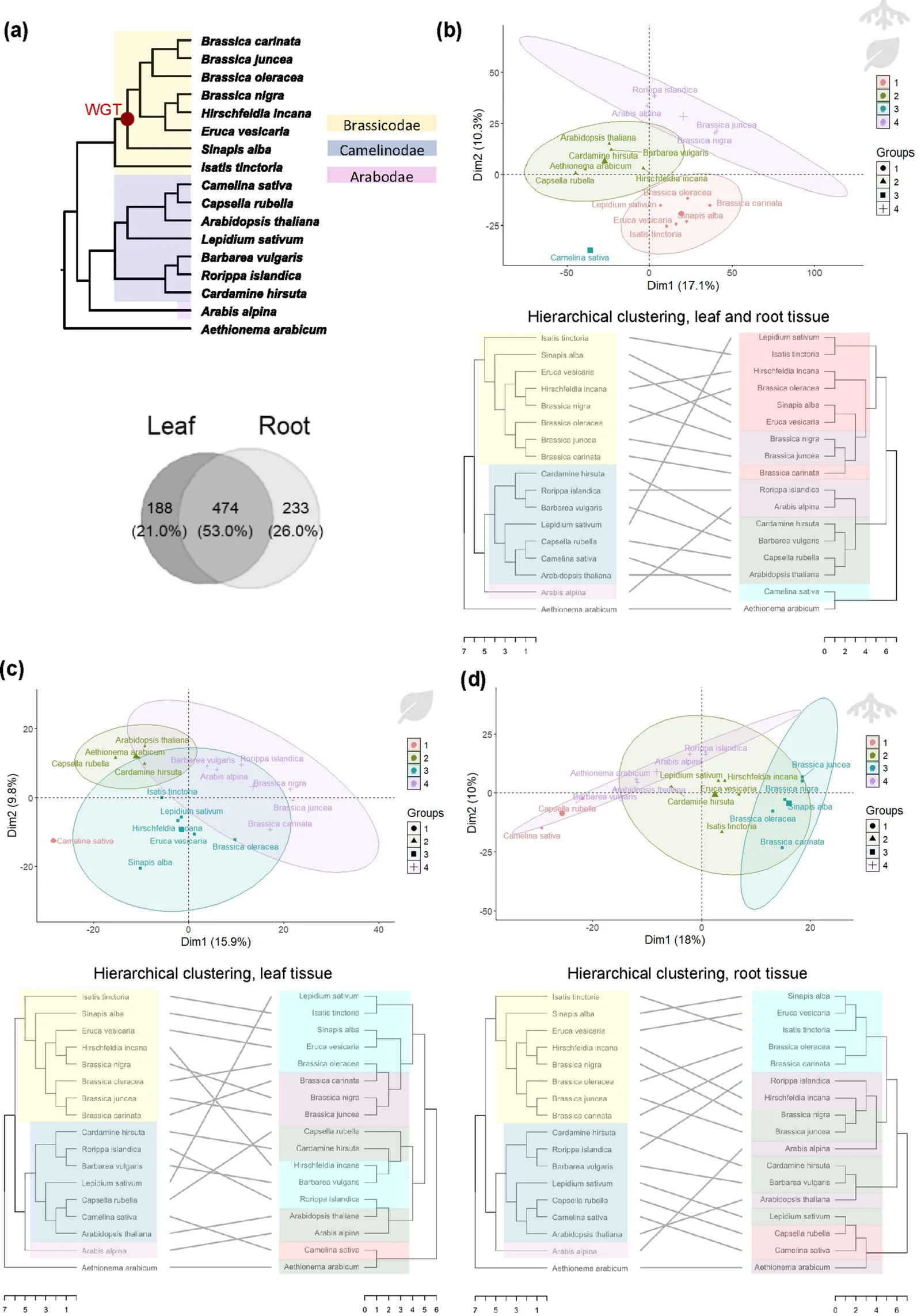
Hierarchical clustering of structural annotations in 17 species across leaf and root tissue and across two batches of LC-MS/MS data. Untargeted LC-MS/MS data was aligned by applying the MS-Cluster algorithm on GNPS2, and subsequently filtering for structural annotations obtained in SIRIUS with a Confidence Exact Score threshold of 0.6. Multiple identical structural annotations potentially resulting from in-source fragmentation and adduct formation were grouped. **a** Phylogenetic tree of species included in hierarchical clustering analysis. WGT, Whole-Genome Triplication event indicated by red circle on the phylogenetic tree. Venn diagram of shared and unique annotations in leaf and root tissue after filtering. **b** PCA and hierarchical clustering of features found across leaf and root tissue. **c** PCA and hierarchical clustering of features found in leaf tissue. **d** PCA and hierarchical clustering of features found in root tissue

LC-MS/MS data of leaf and root tissue extracts from *Rorippa islandica*, *Brassica juncea*, and *Arabis alpina* were integrated as described below with spectra obtained from tissues of 14 species listed in Supplementary Table 1. *A. alpina* represents an additional outgroup to the Camelinodae supertribe, while *R. islandica* and *B. juncea* were added to the Camelinodae and Brassicodae supertribes, respectively (Fig. 6a). Batches of LC-MS/MS data were aligned using the MS-Cluster algorithm within the classical molecular networking workflow in GNPS2. MS-Cluster computes consensus spectra, disregarding retention time shifts across batches. The resulting classical molecular networks of 17 species across leaf and root tissue consist of 14,329 nodes in positive, and 11,271 nodes in negative ionization mode. Filtering for high-confidence annotations computed with SIRIUS v6.0.1 (cutoff: Confidence Exact Score ≥ 0.6) resulted in 1,048 compounds combining positive and negative ionization mode, and across leaf and root tissues of 17 species listed in Supplementary Table 1 as shown in Fig. 6a. Compound structure annotations with confidence exact score ≥ 0.6 obtained in SIRIUS for consensus spectra of mass features were then combined, to merge similar annotations (by matching InChI) across multiple features, resulting in 895 unique structure annotations in a precursor *m/z* range of 130.050 to 1337.720 Da (Material S5).

PCA of all aligned features, across both tissue types, resulted in four clusters, explaining 27.4% of variance in the first two dimensions. Notably, the outgroup is represented by *C. sativa*, while *A. arabicum* clusters with species from the Camelinodae supertribe in PCA cluster 2. Species belonging to the Brassicodae supertribe cluster in two distinct groups, together with *A. alpina* and *Lepidium sativum* in cluster 1 and 4, respectively. The corresponding tanglegram largely aligns with species phylogeny for the complete filtered dataset, including both tissue types, while *L. sativum* and *A. alpina* were found in distinct clusters (Fig. 6b).

Tissue-specific hierarchical clustering of features revealed a similar displacement of *L. sativum* and *A. alpina* for leaf and root tissue. PCA restricted to features found in leaf tissue reveals separation of *Sinapis alba* in cluster 3 from *Brassica* species in cluster 4, primarily along the first dimension explaining 15.9% of variance (Fig. 6c). Notably, PCA of annotated features found in root tissue indicates proximity of *C. sativa* and *C. rubella* in the same cluster, representing the outgroup to the otherwise largely preserved Camelinodae and Brassicodae supertribes (Fig. 6 d). Although clusters for tissue-specific datasets largely overlap, *C. sativa* persistently appears as an outgroup in all three PCA and hierarchical clustering scenarios. In addition, the consistent displacement of *L. sativum* grouping with *I. tinctoria* indicates phytochemical relatedness. Incorporating distinct species in the genus *Lepidium* and *Isatis*, and wild relatives next to domesticated accessions may further resolve tissue-specific chemotaxonomic patterns within the Lepidae tribe.

Our results on comparative chemotaxonomy across two batches of untargeted LC-MS/MS data show that phylogenetic distance can be largely recovered from integrated data across batches, tissues, and ionization modes. Although structure annotation, and hence comparative analysis of compound class distribution is severely limited for this type of analysis, metabolic distance based on hierarchical clustering in the metabolomic tree reflected phylogenetic distance to a large extent. Furthermore, tissue-specific hierarchical clustering highlighted potentially divergent conservation of tissue-specific profiles. Discrete evolutionary pressures on plant tissues have been suspected earlier to result in conserved metabolite profiles incongruent with species phylogeny (Nguyen et al., 2025). When profiles of leaf and root tissue were combined, hierarchical clustering of Brassicodae and Camelinodae species in distinct groups was retained, with two exceptions, namely the placements of *L. sativum* and *C. sativa* in Brassicodae and outgroup, respectively. Further details on the placement of *Brassica* species in all hierarchical clustering scenarios are discussed in Supplementary Material S7. The extension of chemical taxonomy to other supertribes and different plant families may increase the resolution to investigate the relative distance of *C. sativa* to Camelinodae species.

## Outlook and perspectives

To systematically investigate the composition of shared and unique metabolic profiles across the Brassicaceae, we conducted untargeted LC-MS/MS covering 14 species across two major supertribes (Brassicodae and Camelinodae) and two tissues (leaf and root). We assessed taxonomic relationships reflected by conserved metabolite profiles across species and tissues. Our results suggest that comparative metabolic fingerprinting reveals potential hotspots of phytochemical innovation. Current estimates of the total number of unique compounds in the plant kingdom are extrapolated based on a limited number of plant species, while not accounting for phylogenetic distances. We therefore advocate designing studies that systematically profile species across phylogenetic levels to improve estimates of remaining chemical diversity. Balanced, clade-wide sampling can reveal shared biochemical traits within lineages and help correct biases toward model and crop species. Furthermore, we established a computational pipeline for pre-processing and annotation of positive and negative ionization mode LC-MS/MS data integrating in silico structure, substructure, and compound class annotation tools. We found that library matches and consensus predictions on chemical ontology levels overlap with predicted substructures (Mass2Motifs), highlighted for the triterpenoid compound class. Our results also align with previously described trends in phytochemical diversity across multiple species, as found for alkaloids and triterpenoid saponins in *I. tinctoria* and *B. vulgaris*, respectively.

Since specialized metabolites are often stress-induced, metabolic fingerprinting reflects only a snapshot of plant chemical diversity, underscoring the importance of systematically eliciting hormone-induced stress responses in experimental designs to further probe the plant biochemical repertoire. Furthermore, we note that our analytical approach does not provide unambiguous proof of structural identities; however, our framework was designed to combine inputs from multiple different sources to provide consensus annotations, making annotations on chemical ontology levels more reliable. Ultimately, our experimental and computational workflows provide a modular and extendible framework for systematic metabolic fingerprinting to study the evolution of plant natural product biosynthesis.

## Materials and methods

### Plant material and growth conditions

Seeds of plant genotypes listed in Supplementary Table 1 were vapor-sterilized using chlorine gas in a desiccator for 3 h, cold stratified at 8 °C for seven days, and sown on half-strength Murashige and Skoog (MS) agar. Plants were grown for 25 to 35 days in a controlled growth chamber (16 h : 8 h, light : dark photoperiod, 140–180 µmol m2 s-1, 22 ± 2 °C) until seedlings developed four mature leaves (PO:0007115). Plant positions in the climate chamber were randomized.

### Plant tissue sampling

Plants were harvested from agar pots, and leaf and root tissue were carefully separated, using a scalpel, excluding stem and cotyledons. Residual growth medium was removed from roots by washing with demineralized water. Harvesting was performed after eight to 10 h of light exposure (between 13:00 h and 15:00 h), and collected tissues were immediately frozen in liquid nitrogen. Samples from three biological replicates per species consisting of five to 20 plants per replicate grown in independent tissue culture units were harvested and homogenized under liquid nitrogen using a mortar and pestle and stored at −80 °C until further analysis.

### Metabolite extraction and LC-MS/MS analysis

Tissue samples were processed for metabolite extraction and analysis according to De Vos et al. (2007). Briefly, solvent (99.875% methanol acidified with 1.25% formic acid) was added to fresh tissue material in a 1:3 ratio (fresh weight tissue to solvent), followed by sonication for 15 min at 40 kHz in a water bath at 20 °C. The liquid phase was obtained after centrifugation at 15,000 g. Samples were randomly injected (volume: 10 µl) at 20 °C into a Vanquish UHPLC with Exploris120 Orbitrap system (Thermo Fisher Scientific, Waltham, MA, USA). The used Vanquish UHPLC was equipped with an Vanquish photodiode array detector (220–600 nm) connected to an Orbitrap Exploris 120 mass spectrometer fitted with an electrospray ionization (ESI) source. A reverse-phase column (Acquity UPLC BEH C18, 1.7 μm, 2.1 × 150 mm; Waters) was used at 40 °C for chromatographic separation. Samples were analyzed with a flow rate of 0.4 ml/min with stepwise peak ratios of mobile phase eluents A (0.1% formic acid in ultrapure water), and B (0.1% formic acid in acetonitrile) with initial conditions set to 95:5 (A: B). After the linear gradient of 22 min (0.0–22.0 min, 5–75% B), washout phases (1 min gradient 75% to 90% B, 3 min at 90% B) were followed by recalibration to initial conditions (95%A:5%B). Scans were obtained with an *m/z* range of 90–1350 Da in positive and negative mode (switched polarity) and a resolution of 60,000 FWMH. Data was obtained in DDA mode (centroid, Number of Dependent Scans = 4) with normalized HCD Collision Energies (%) = 20,40,100. Mass exclusion lists (Material S1) were generated from solvent blank samples injected prior to the tissue sample extracts, to avoid fragmentation of mass features present in the solvent under DDA mode. Quality Control (QC) samples were prepared from pooled samples, including material from all analyzed samples. Further details on data acquisition, corresponding metadata, and preprocessing can be found in Wolters et al. (2026). Due to the exploratory nature of this untargeted metabolomics study covering multiple species, QC samples were used instead of internal standards. To stabilize and calibrate the analytical platform, multiple QC injections were performed prior to the analysis of the plant tissue samples.

### LC-MS/MS data processing

Raw Waters MS data was converted to .*mzXML* file format using MSConvert v3.0. Extraction of metabolite features was conducted in mzmine v4.8.0 (Schmid et al., 2023) for a Retention Time (RT) window between 0.8 and 22.8 min. Presets are provided as.*mzbatch* file in XML format (Material S2). Aligned feature lists generated in mzmine were exported in MGF spectra format, and feature quantification tables in.csv format. Feature lists generated for spectral data of 14 species, and mzXML files of the combined dataset including spectral data of 17 species were submitted to the Global Natural Products Social (GNPS) Molecular Networking online platform using the Feature-Based Molecular Networking (FBMN) and Classical Molecular Networking (CMN) workflows, respectively (GNPS2 Release 2025.12.10, Wang et al., 2016). Intermediate processing files and settings for feature extraction are available on Zenodo (DOI: https://doi.org/10.5281/zenodo.19391098).

### Structure and compound class annotation

Extracted features of 14 species were annotated in MS2Query v1.0 (de Jonge et al., 2023), DreaMS v1.0 (Bushuiev et al., 2025), and in SIRIUS v6.1.0 (Dührkop et al., 2019), including assessment of prediction accuracy in ZODIAC (Ludwig et al., 2019), and structure annotation by CSI: FingerID (Dührkop et al., 2015). Spectral library searches using DreaMS v1.0 utilized the MassSpecGym spectral library (Bushuiev et al., 2024). Compound class annotations based on NP.Classifier (Kim et al., 2021) were obtained in CANOPUS (Dührkop et al., 2021) for fingerprints computed in SIRIUS v6.1.0, and for analog predictions in MS2Query v1.0. NP.Classifier annotations were computed via the API (v1.5) for all structure matches and analog predictions obtained with DreaMS v1.0 regardless of similarity scores. Consensus structure and chemical ontology predictions were computed separately for exact matches of InChI key and compound class strings obtained in SIRIUS v6.1.0 and CANOPUS, and converted from SMILES obtained in DreaMS v1.0 and MS2Query v1.0, using an in-house script available on GitHub (https://github.com/capsicumbaccatum/Manuscript_Metabolic_profiling).

Total number, compound class categories and overlap of annotations per annotation tool are available on Zenodo (DOI: https://doi.org/10.5281/zenodo.19391098) and visualized in Supplementary Fig. S4. The combined output of metabolite feature annotations and consensus predictions was mapped on the FBMN. The consensus spectra of the CMN obtained for the combined dataset including 17 species was annotated with SIRIUS v6.1.0, using a cutoff of 0.6 for the Exact Confidence Score in downstream analysis.

### Mass2Motif analysis

Mass2motifs were obtained with MS2LDA v2.0.1 (van der Hooft et al., 2016; Wandy et al., 2018; Torres-Ortega et al., 2025), with settings provided in Material S3. In short, the number of discovered motifs was set to 399, and the number of iterations to 1000, using default settings for all other parameters. Mass2motifs were then mapped onto the FBMN with a doc-topic probability score threshold of 0.5 and an overlap score threshold of 0.15.

### Hierarchical clustering, PCA and chemical taxonomy analysis

Hierarchical clustering was computed for a distance matrix generated by the *dist()* function, specifying Euclidean distance. The *hclust* object was computed based on complete clustering (R package *stats* v4.5.1, R Core Team, 2016) and subsequently converted to a dendrogram object for plotting against species phylogeny (according to Hendriks et al., 2023) using the *tanglegram* function of the R package *dendextend* v1.19.1. Tanglegrams were constructed for the full set of features in the CMN consensus MGF, for a subset of annotated features using SIRIUS v6.0.1 structure predictions with a Confidence Exact Score threshold of 0.6, and for features specific to leaf and root tissue, respectively. Based on the alignment with species phylogeny, tanglegrams are presented for comparative analysis between tissue types.

### Data visualization and formatting

Analyses and visualizations conducted in R v4.5.1 (R Core Team, 2016 ) employed the following packages: DPLYR (Wickham et al., 2023) and TIDYVERSE (Wickham et al., 2019) for data formatting and filtering; GGPLOT2 (Schloerke et al., 2021), GGTREE and GGTREEXTRA (Xu et al., 2021, 2022), COMPLEXUPSET (Krassowski et al., 2022) and VENN (Ruskey & Weston, 2005) for visualization and annotation of phylogenetic trees and upset plots. Analyses and visualizations conducted in Python v3.12.0 employed the following packages: RDKit v2025_09_3 (Landrum et al., 2025) for SMILES string conversion and Matchms (Huber et al., 2020) and MS2LDA 2.0 (Torres Ortega et al., 2025) for visualization of mass spectra, motif extraction and formatting.

## Supplementary Information

Below is the link to the electronic supplementary material.

Supplementary material 1 (PDF 4887.8 kb)

Supplementary material 2 (DOCX 12.2 kb)

## Data Availability

The LC-MS/MS data collected in this study has been submitted to MetaboLights (MTBLS14270) and MassIVE (MSV000101479). Scripts are available on Github (https://github.com/capsicumbaccatum/Metabolic_fingerprinting_consensus_annotation). Supplementary data, such as intermediate results can be found on Zenodo (DOI:10.5281/zenodo.19391098).
