# Supplementary material for: Systematic mass-spectrometry-guided metabolic fingerprinting elucidates diversity of specialized metabolites across the Brassicaceae
Source: Metabolomics. 2026 Sep 25;22(5):161. doi: 10.1007/s11306-026-02537-y (PMC13615159; doi:10.1007/s11306-026-02537-y)
Supplement: Supplementary file 1 [file 11306_2026_2537_MOESM1_ESM.pdf]

Supplementary Material S7

Journal: *Metabolomics*

Systematic mass-spectrometry-guided metabolic fingerprinting elucidates diversity of specialized metabolites across the Brassicaceae

Felicia C. Wolters[1,2], Tina Woldu[1,2], M. Eric Schranz[2], Marnix H. Medema[1], Klaas Bouwmeester[2,†], Justin J. J. van der Hooft[1,3,†]

1. Bioinformatics Group, Wageningen University & Research, 6708PB, Wageningen, the Netherlands
2. Biosystematics Group, Wageningen University & Research, 6708PB, Wageningen, the Netherlands
3. Department of Biochemistry, University of Johannesburg, 2006 Johannesburg, South Africa

[†]Shared last authors
Author for correspondence:
Felicia Wolters, Klaas Bouwmeester, Justin J. J. van der Hooft
Tel: +31 6 44 55 84 99
Emails: felicia.wolters@wur.nl, klaas.bouwmeester@wur.nl, justin.vanderhooft@wur.nl

Contents

Characteristic triterpnoid saponin-like fragmentation patterns in negative ionization mode…S1

Root and leaf tissue profiles……………………………………………………………………..S2

Distribution of unique features in Camelinodae species……………………………………...S3

Chemical taxonomy of 17 species on distinct classification levels…………………………..S4

Hierarchical clustering of Brassica species…………………………………………………..S5

Figures

Figure S1: Distribution of mass features across leaf and root tissue…………………………..S6

Figure S2: Distribution of metabolite features exclusively found in leaf and root tissue……..S7

Figure S3: Distribution of metabolite features annotated as alkaloids…………….……..….S8

Figure S4: Total number and overlap of computational annotation tools……………………..S9

Figure S5: Distribution of triterpenoid consensus superclass annotations………….……....S10

Figure S6**:** Mappings of Mass2Motifs in negative ionization mode…………………………S11

Figure S7: Structural annotations in 17 species across leaf and root tissue………………...S12

Figure S8: Hierarchical clustering of aligned filtered and unfiltered metabolite features…..S13

Tables

Table S1: Used plant species and genotypes organized by taxonomic order……………….S14

Table S2: Number of metabolite features obtained for integrated batches………………….S15

The "core-metabolome" is substantially different across species within supertribes

The shared sets of metabolite features across species are characterized by annotations for primary metabolite classes, such as amino acids and peptides, carbohydrates, and fatty acids.

Within the Camelinodae supertribe, species share in total more than 200 features, predominantly annotated as terpenoids, alkaloids or shikimates and phenylpropanoids in positive ionization mode. The number of shared metabolite annotations across sets of species within the Camelinodae reflects taxonomic relationships, with *Camelina sativa* and *Capsella rubella* sharing 75 (3.8%) annotated features, followed by *C. sativa* and *Arabidopsis thaliana* (42, 2.3%) in positive ionization mode (Fig. 2a). In contrast, species of the Brassicodae supertribe share smaller sets with less than 20 features (2%) under positive ionization mode (Fig. 2a). In negative ionization mode, *Brassica carinata* shares 23 (3.3%) features primarily annotated as amino acids and peptides with *Brassica nigra*, and 25 (3.4%) features with *Brassica oleracea,* mostly annotated as shikimates and phenylpropanoids (Fig. 2b). All other species of the Brassicodae supertribe have less than 20 metabolite features with consistent NPC.pathway annotations in common (Material S6).

Root and leaf tissue show distinct degrees of conservation in metabolic fingerprints

To investigate tissue-specific conservation and composition across supertribes and species, compound class annotations were assessed in leaf and root tissue profiles across supertribes and species. Positive and negative ionization modes revealed distinct patterns of specialized metabolite class distribution in both tissue types. In positive ionization mode, 2,206 features were identified in leaf tissue and 1,675 features in root tissue across all 14 species, with 1,237 features shared between both tissues (Fig. S1). In negative ionization mode, 1,878 metabolite features were leaf-specific, and 1,327 were root-specific, while 902 were extracted from both tissues (Fig. S1b). The largest number of unique metabolite features exclusively obtained from root tissue was found in *B. vulgaris* in negative ionization mode (207, 59.7%, Fig S1b2). Notably, the root tissue profile of *B. vulgaris* exhibited a higher proportion of unique metabolite features than in leaf tissue in both ionization modes, whereas most other species showed the opposite trend (Fig. S1). The shared metabolite profile across all species displays a comparable composition of compound classes across tissues and ionization modes, representing 4.2% of all detected features in leaf and in root under positive mode, and 2.2% in leaf and 2.6% in root

under negative mode (Fig. S1). Similarly, on the level of supertribes, tissue profiles shared between Camelinodae, Brassicodae and outgroup are comparable in positive (535 features, 15.4% in leaf and 477 features, 16.4% in root) and negative (285 features, 10.2% in leaf, 288 features, 13.0% in root) ionization modes. Adding the dimension of tissue revealed distinct degrees of conservation in species. The largest number (68, 5.4%) of metabolite features shared by two species was found in the Camelinodae (*C. sativa* and *C. rubella*) in root tissue under positive ionization mode, dominated by alkaloid and terpenoid annotations. In contrast, the shared metabolite features between *B. carinata*, *B. nigra* and *B. oleracea* belong to the leaf tissue profiles under negative ionization mode, dominated by amino acids, peptides, shikimates and phenylpropanoids (Fig. S1b1).

Our results indicate tissue-specific conservation of metabolic fingerprints in related plant species, underscoring the importance of spatial variation, and its implications for gene regulation in plant specialized metabolite biosynthesis. However, we suggest to interpret observed variation in leaf and root tissue metabolomes in the context of plant ontogeny, as recently highlighted for *Solanum dulcamara* (Anaia *et al*., 2025). Hence, metabolic fingerprinting may reveal distinct patterns of phytochemical relatedness across tissues depending on plant developmental stages.

Table 1: Used plant species and genotypes organized by taxonomic order

Supertribe§	Tribe§	Genus	Species	Accession code
Brassicodae	Brassiceae	*Brassica* (*Mutarda*)	*nigra* †	SF48
Brassicodae	Brassiceae	*Hirschfeldia*	*incana*†	Nijmegen
Brassicodae	Brassiceae	*Eruca*	*vesicaria* †	PI 6333218
Brassicodae	Brassiceae	*Brassica*	*carinata* †	Gomenzer
Brassicodae	Brassiceae	*Brassica*	*juncea* ‡	CGN15183
Brassicodae	Brassiceae	*Brassica*	*oleracea* †	CGN18947
Brassicodae	Brassiceae	*Sinapis*	*alba* †	S2 GC0560-79
Brassicodae	Isatidae	*Isatis*	*tinctoria* †	Weber
Camelinodae	Camelineae I	*Camelina*	*sativa* †	Daan-15
Camelinodae	Camelineae I	*Capsella*	*rubella* †	Monte Gargano
Camelinodae	Arabidopsideae	*Arabidopsis*	*thaliana* †	Col-0
Camelinodae	Lepideae	*Lepidium*	*sativum* †	SluisG
Camelinodae	Cardamineae	*Barbarea*	*vulgaris* †	Zodiac
Camelinodae	Cardamineae	*Rorippa*	*islandica* ‡	GC 2006-89
Camelinodae	Cardamineae	*Cardamine*	*hirsuta* †	De Hoef
Arabodae	Arabideae	*Arabis*	*alpina* ‡	Pajares
Outgroup	Aethionemeae	*Aethionema*	*arabicum* †	ES1020

†Included in batch A (14 species); ‡Included in batch B (3 species); §Phylogeny according to Hendriks *et al.* (2023)[5].

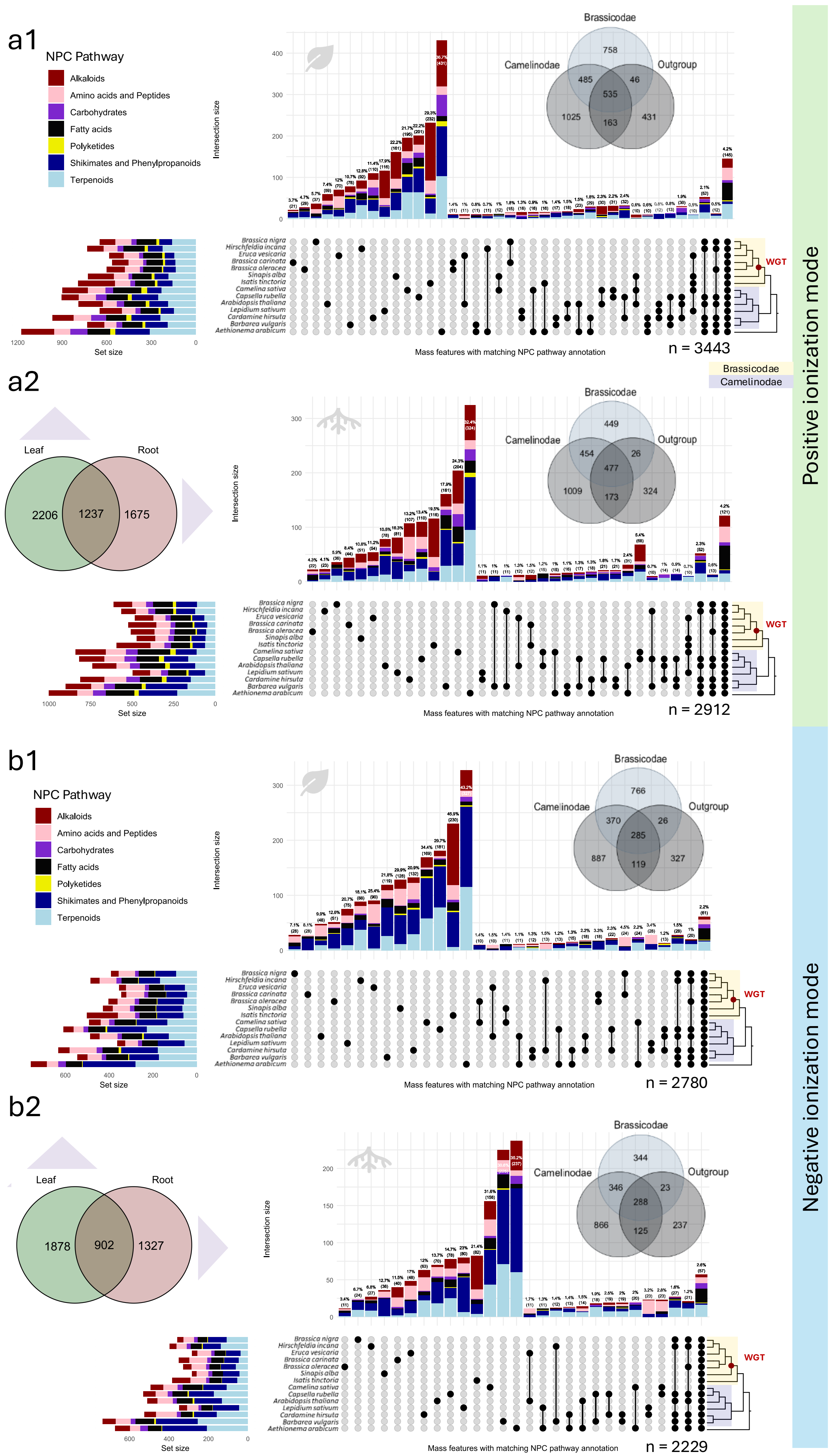
a1
NPC Pathway
Alkaloids
Amino acids and Peptides
Carbohydrates
Fatty acids
Polyketides
Shikimates and Phenylpropanoids
Terpenoids
Intersection size
Brassicodae
Camelinodae
Outgroup
758
485
46
535
1025
163
431
Set size
Mass features with matching NPC pathway annotation
n = 3443
WGT
Brassicodae
Camelinodae
a2
Leaf
Root
2206
1237
1675
449
454
26
477
1009
173
324
n = 2912
b1
766
370
26
285
887
119
327
n = 2780
b2
1878
902
1327
344
346
23
288
866
125
237
n = 2229
Positive ionization mode
Negative ionization mode
Brassica nigra
Hirschfeldia incana
Eruca vesicaria
Brassica carinata
Brassica oleracea
Sinapis alba
Isatis tinctoria
Camelina sativa
Capsella rubella
Arabidopsis thaliana
Lepidium sativum
Cardamine hirsuta
Barbarea vulgaris
Aethionema arabicum

Figure S1: Distribution of mass features across leaf and root tissue in 14 Brassicaceae species. Untargeted metabolomics (LC-MS/MS) data was filtered for consistent annotations on the NP.Classifier "pathway" chemical ontology level. Red dots on phylogenetic trees indicate the Whole Genome Triplication (WGT) event in the Brassicodae clade. Venn diagrams show overlaps of aligned features across supertribes. **(a)** Metabolite features annotated in positive ionization mode in (**a1**) leaf and (**a2**) root tissue. **(b)** Metabolite features annotated in negative ionization mode in (**b1**) leaf and (**b2**) root tissue.

Metabolite profiles of *Camelina sativa*, *Isatis tinctoria*, *Capsella rubella*, and *Barbarea vulgaris* contain large numbers of unique features

To investigate the size and composition of unique metabolite profiles across species, we compared the proportions of annotated compound classes in the "accessory" (unique) metabolome. In total, between 7% and 49.5% of detected metabolite features were found to be unique per species. Across all species, unique metabolite profile annotations were enriched with alkaloids (27.96%), shikimates and phenylpropanoids (25.54%), and terpenoids (19.07%) in positive ionization mode. In negative ionization mode, enrichment with shikimates and phenylpropanoids (37.92%), and with terpenoids (23.48%) was found higher than with alkaloids (15.23%) in annotated unique metabolite profiles. Across both ionization modes, the outgroup species *A. arabicum* exhibited the highest number of unique features with matching NP.Classifier annotations, with 688 (44.3%) metabolite features in positive ionization mode, and 504 (49.5%) metabolite features in negative ionization mode (Fig. 2a & 2b). In positive ionization mode, *Camelina sativa* showed the highest total number of unique mass features (387, 30.2%) within the Camelinodae, followed by *Isatis tinctoria* (325, 33%) in the Brassicodae. Notably, *I. tinctoria* (Isatidae tribe) also possessed the largest number of unique alkaloid pathway annotations across all intersections and in both ionization modes, resembling taxonomic distance to species in the Brassiceae sister tribe (Fig. 2a & 2b).

In positive and negative ionization mode, alkaloid profiles were particularly enriched in tryptophan alkaloid superclass annotations across all species, and anthracillic acid alkaloids specifically in I. *tinctoria*, while ornithine alkaloids were mostly abundant in *A. arabicum* in positive ionization mode (Fig. S4).

In negative ionization mode, *Barbarea vulgaris* displayed the largest unique profile of annotated mass features (326, 36.5%) in the Camelinodae. However, corrected for the total set size (total number of detected metabolite features) per species, *I. tinctoria* showed the largest proportion of unique features in positive (33%) and negative (45.1%) ionization mode (Fig.

2b). In summary, the accessory metabolite profiles of three species in the Camelinodae supertribe (*B. vulgaris, C. sativa, C. rubella*) and in *I. tinctoria* (Brassicodae) are outstanding, with terpenoid profiles disproportionally enriched in Camelinodae species.

The outstanding unique metabolite profiles of *I. tinctoria* and *B. vulgaris* were found in alignment with previous research focusing particularly on alkaloid and terpenoid diversity in *I. tinctoria (Xu et al., 2020)* and *B. vulgaris* (Erthmann *et al*., 2018; Shen *et al*., 2025; Shinoda *et al*., 2002), respectively. Furthermore, our results suggest that the metabolite profile of *C. sativa* is particularly distinctive, also representing the largest terpenoid profile next to the outgroup *A. arabicum* in positive ionization mode (Fig. 2a). The evolutionary history of *C. sativa* may partially explain its outstanding unique metabolite profile. *C. sativa* has undergone several rearrangements of its hexaploid genome (*n*=20), which arose from hybridization between a *C. neglecta*-like genome (*n*=6) and *C. hispida* (*n*=7) (Mach *et al.,* 2019). The characterization of the metabolite profile of *C. sativa* has been mostly focused on mono- and sesquiterpenoids (Augustin *et al.,* 2019), amino acids, flavonols, cinnamic acids and glucosinolates in previous research (Alberghini *et al*., 2022; Guendouz *et al*., 2022; Zhang & Parkin, 2025). Our findings suggest that the terpenoid profile of *C. sativa* constitutes a diverse mixture of terpenoid classes, including triterpenoids, and is outstanding and unique in the panel of species included in our analysis. While the high content of polyamines in *C. sativa* has been linked earlier to advanced resilience against abiotic stress (Zhang & Parkin, 2025), terpenoid diversity has not yet been studied in this species in greater detail to our knowledge, especially with regards to high-mass compounds (> m/z 1000). The number of unique features in the terpenoid NPC.pathway consistently annotated in *C. sativa* (92, 33.5%) under positive ionization mode is about two-fold higher than in *B. vulgaris* (42, 17.4%), while the latter is known to produce a diverse range of specific terpenoids (Khakimov *et al.,* 2016). Studying metabolite profiles of *C. sativa* progenitors (*C. neglecta* and *C. hispida*) may elucidate the impact of genome rearrangements following polyploidization in *C. sativa*, which has potentially resulted in the diversification of terpenoid biosynthesis.

(a)

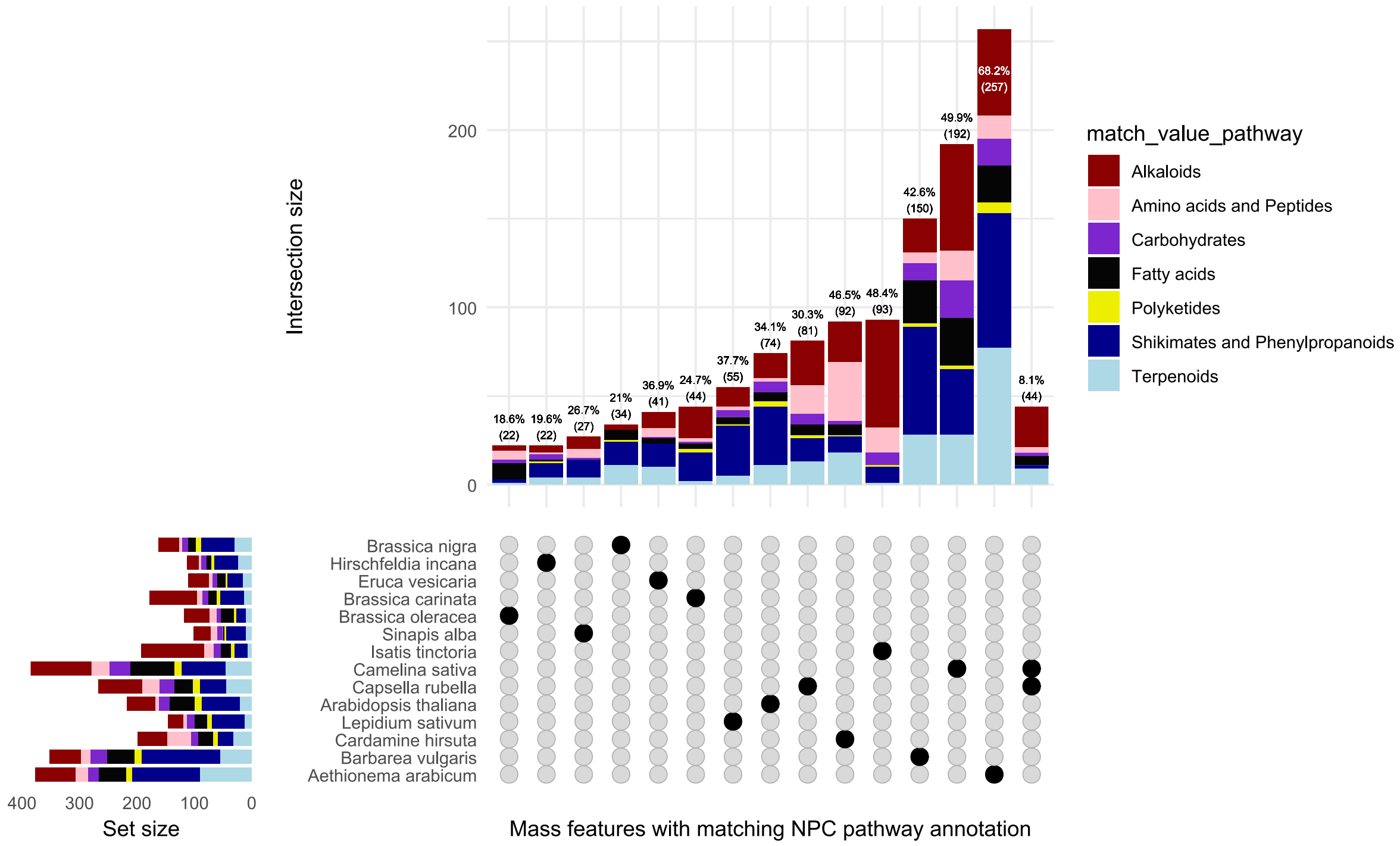
Intersection size
200
100
0
match_value_pathway
Alkaloids
Amino acids and Peptides
Carbohydrates
Fatty acids
Polyketides
Shikimates and Phenylpropanoids
Terpenoids
Brassica nigra
Hirschfeldia incana
Eruca vesicaria
Brassica carinata
Brassica oleracea
Sinapis alba
Isatis tinctoria
Camelina sativa
Capsella rubella
Arabidopsis thaliana
Lepidium sativum
Cardamine hirsuta
Barbarea vulgaris
Aethionema arabicum
400 300 200 100 0
Set size
Mass features with matching NPC pathway annotation

(b)

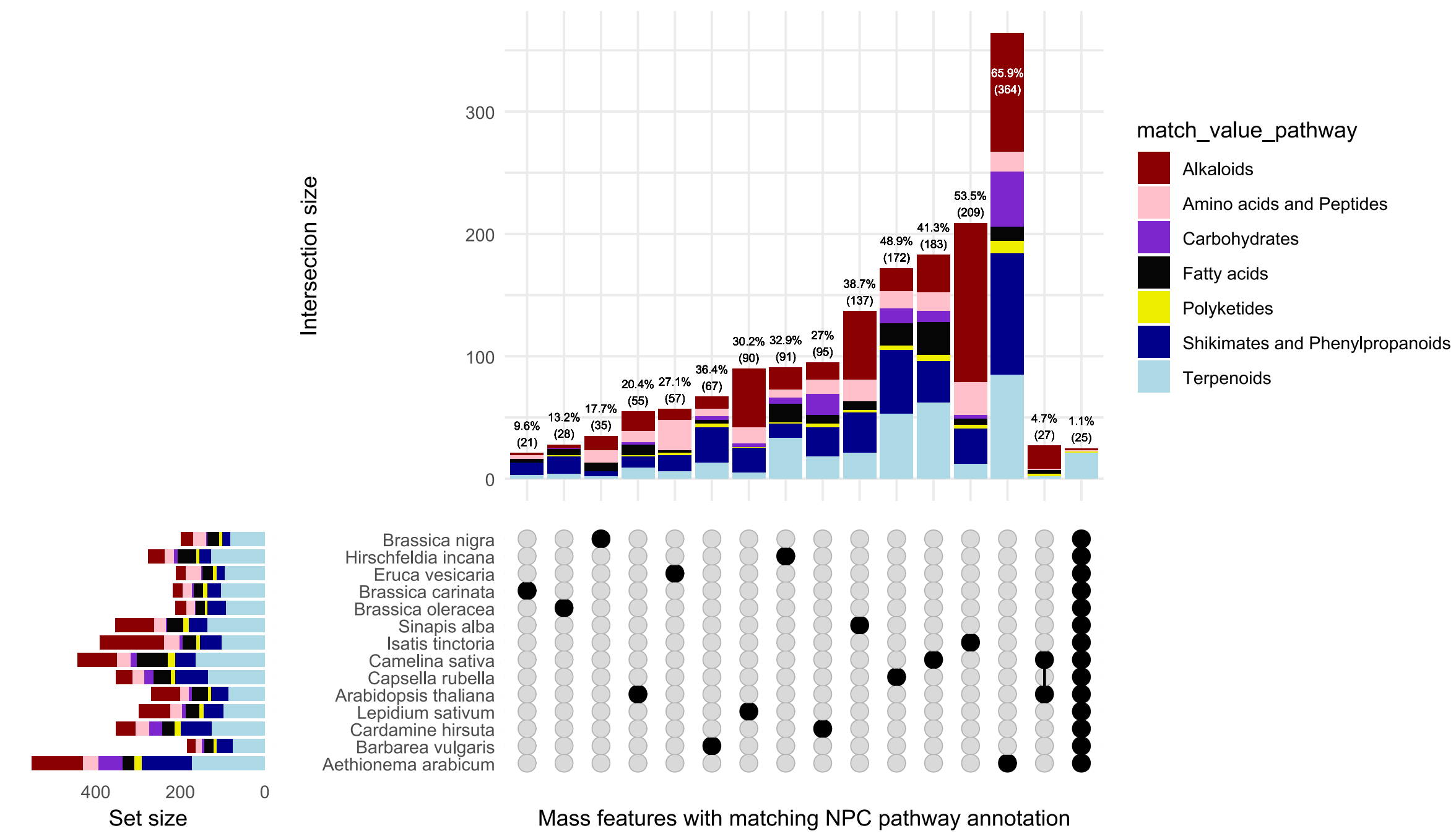
Intersection size
300
200
100
0
match_value_pathway
Alkaloids
Amino acids and Peptides
Carbohydrates
Fatty acids
Polyketides
Shikimates and Phenylpropanoids
Terpenoids
Brassica nigra
Hirschfeldia incana
Eruca vesicaria
Brassica carinata
Brassica oleracea
Sinapis alba
Isatis tinctoria
Camelina sativa
Capsella rubella
Arabidopsis thaliana
Lepidium sativum
Cardamine hirsuta
Barbarea vulgaris
Aethionema arabicum
400 200 0
Set size
Mass features with matching NPC pathway annotation

(c)

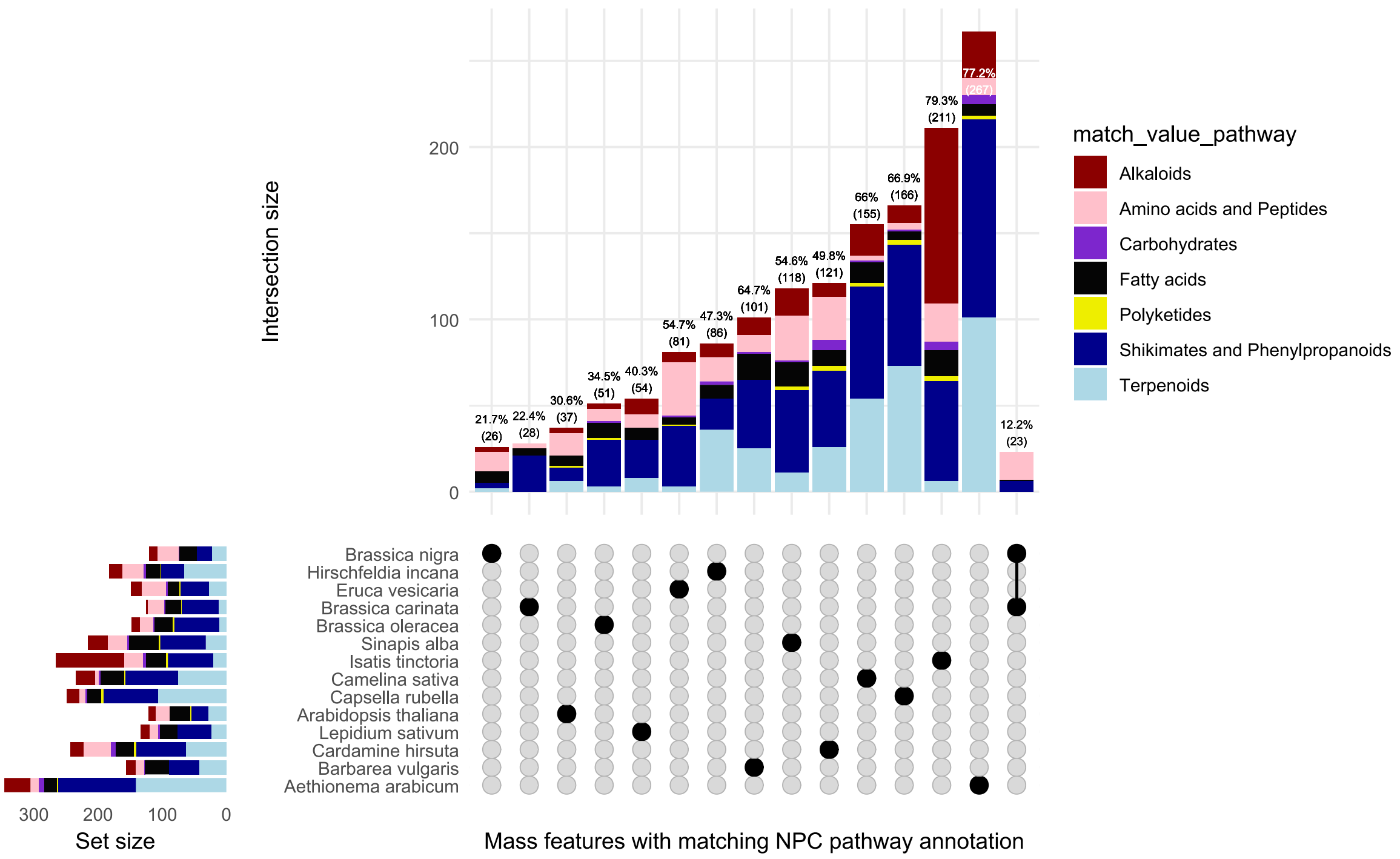

(d)

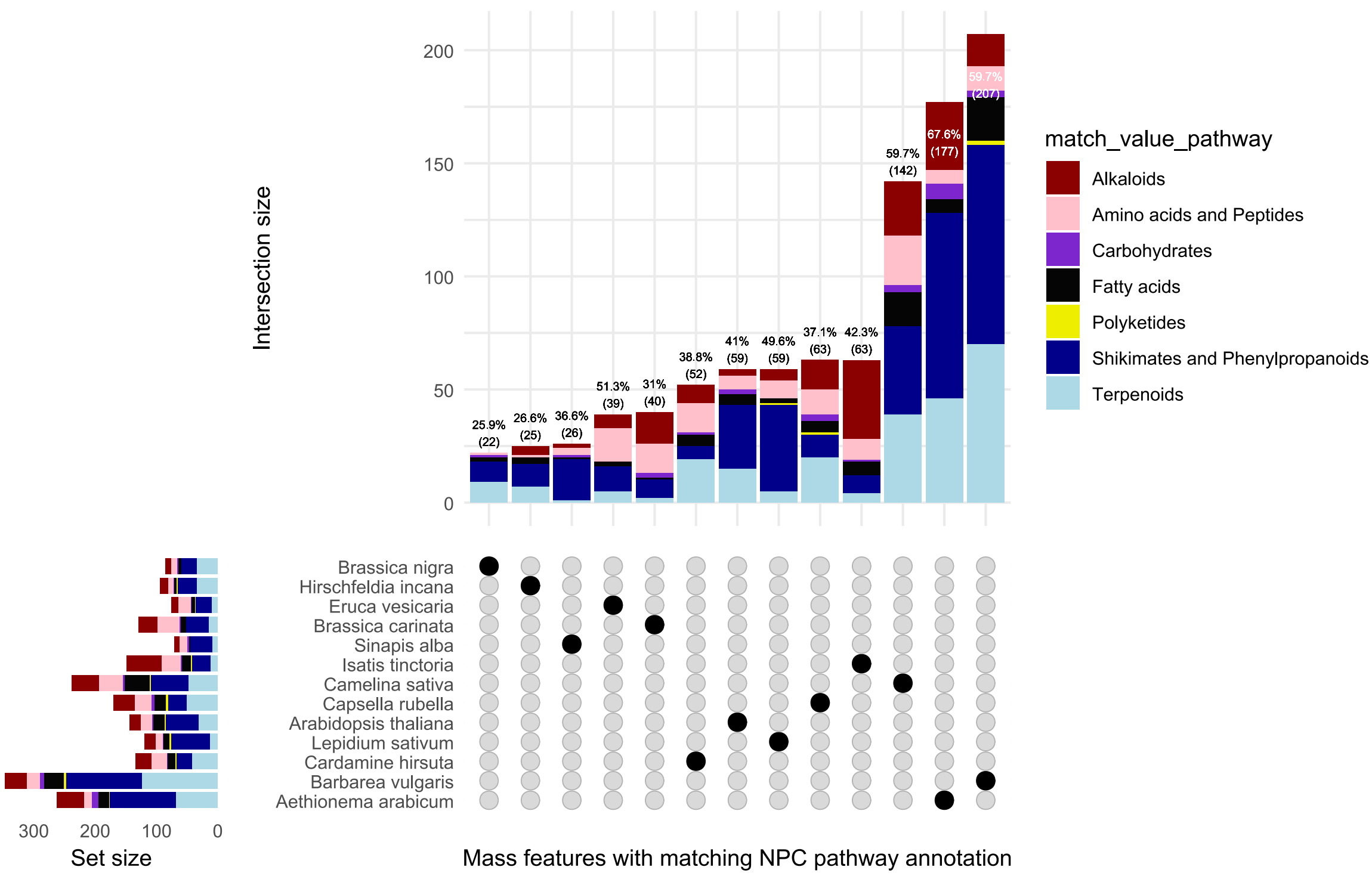

Figure S2: Upset plots showing shared and unique annotated metabolite features exclusively found in leaf and root tissue of 14 species. **(a)** Metabolite features detected exclusively in leaf tissue under positive ionization mode. **(b)** Metabolite features detected exclusively in root tissue under positive ionization mode. **(c)** Metabolite features detected exclusively in leaf tissue under negative ionization mode. **(d)** Metabolite features detected exclusively in root tissue under negative ionization mode.

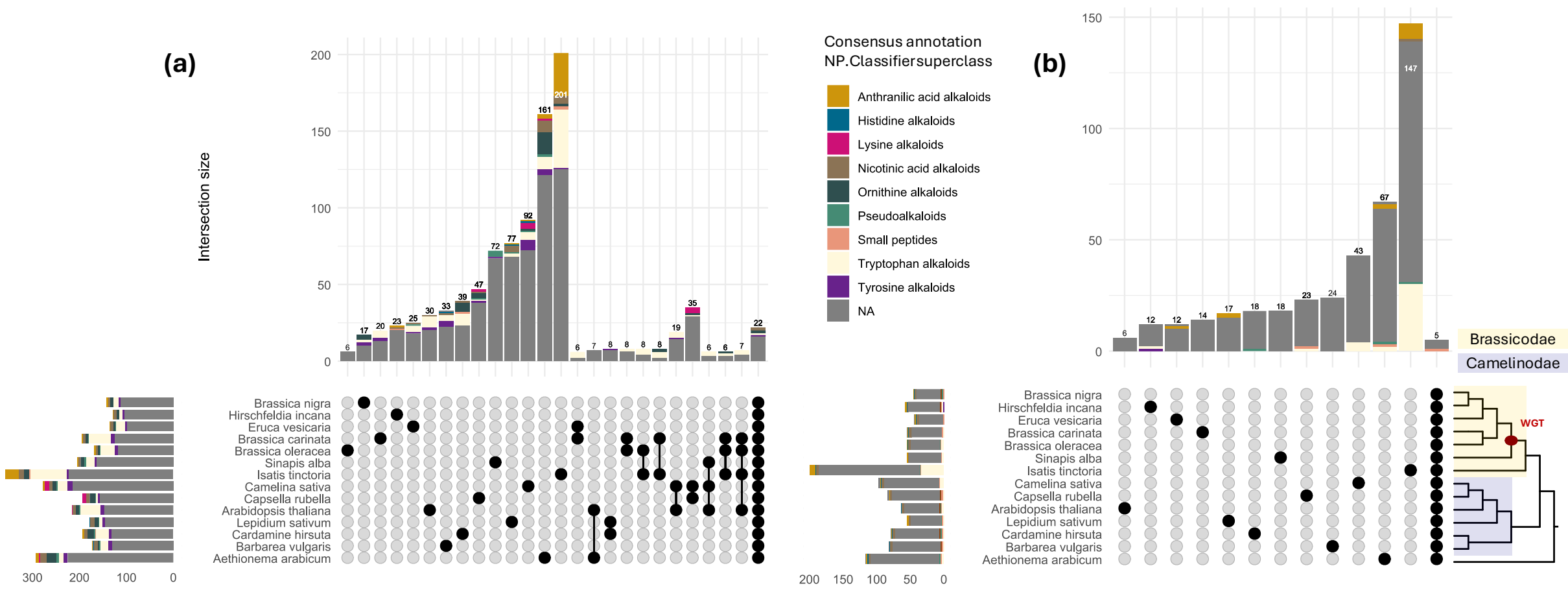

Figure S3: Distribution of mass features consistently annotated as alkaloids on the NP.Classifier chemical "pathway" ontology level across 14 Brassicaceae species under positive and negative ionization mode. Untargeted metabolomics (LC-MS/MS) data was filtered for consistent annotations using computational annotation tools (SIRIUS, MS2Query, DreaMS) on the NP.Classifier superclass chemical ontology level, filtered for "alkaloid" pathway annotations. Alkaloid pathway annotations with inconsistent superclass annotation across at least two annotation tools are indicated in grey ("no match"). The red dots placed on the phylogenetic tree indicate the Whole Genome Triplication (WGT) event in the Brassicodae clade. (a): Metabolite features annotated in positive ionization mode. (b): Metabolite features annotated in negative ionization mode.

Distribution of compound class annotations on chemical ontology levels by NP.Classifier

(a)

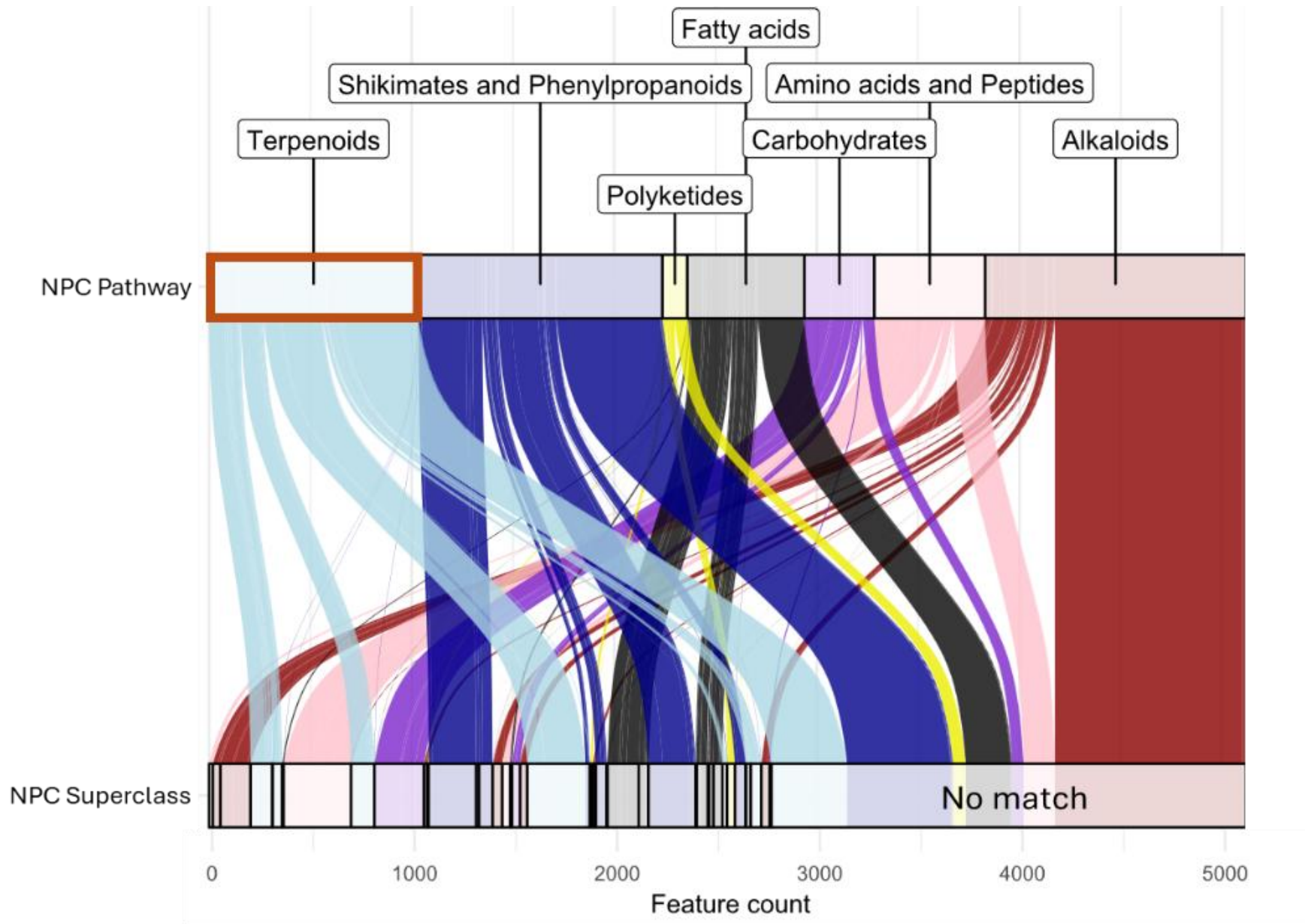

Consensus annotation

- Pathway (all) 5,118
- Pathway: Terpenoids 1,032
- Superclass: Triterpenoids 108

(b)

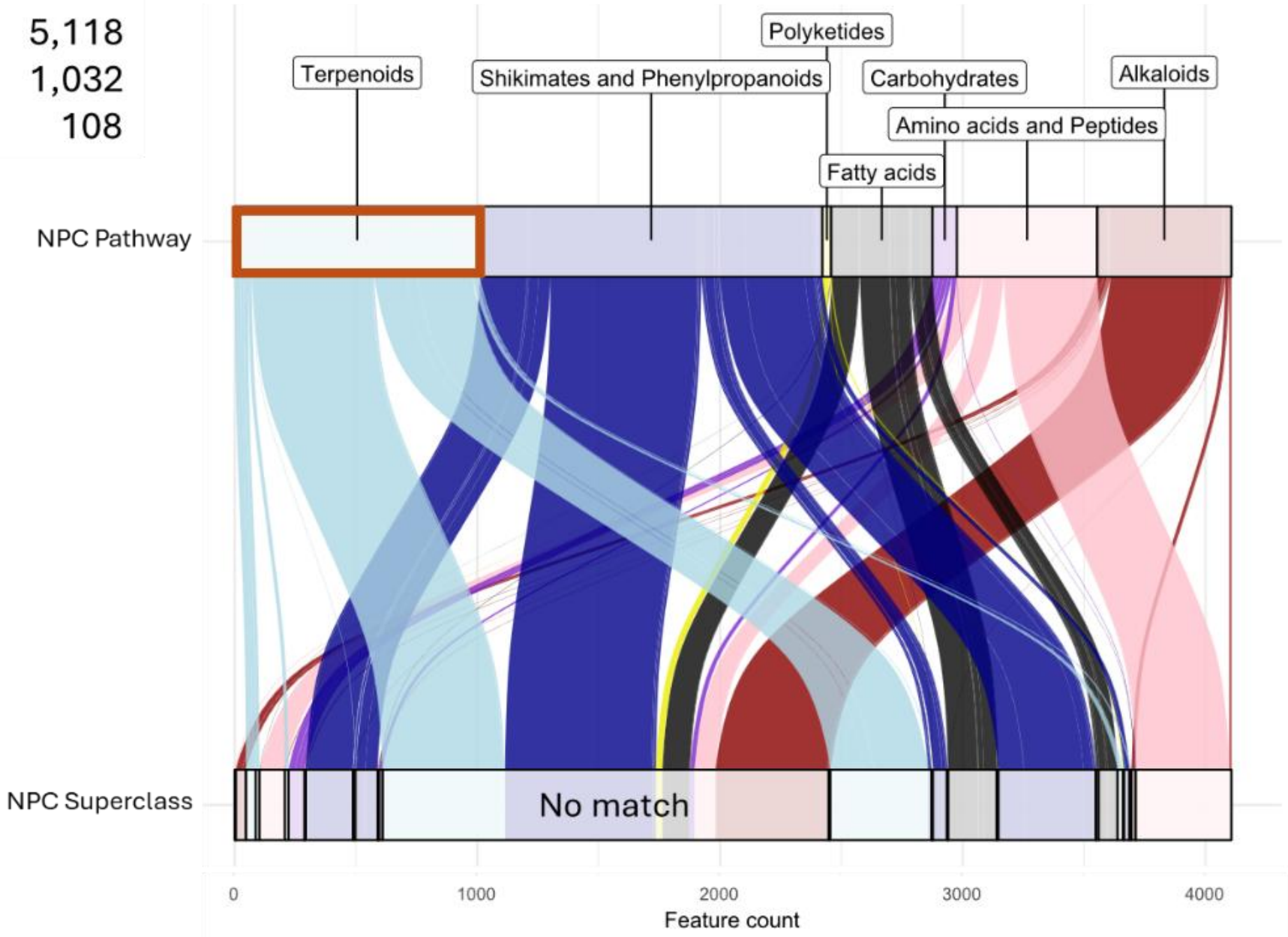

Consensus annotation

- Pathway (all) 4,107
- Pathway: Terpenoids 1,011
- Superclass: Triterpenoids 40

Figure S4: Total number and overlap of computational annotation tools (SIRIUS v6.1.0, MS2Query v1.0 and Dreams v1.0 against the MassSpecGym library) across 14 species and two tissues (leaf and root) in **(a)** positive and **(b)** negative ionization mode.

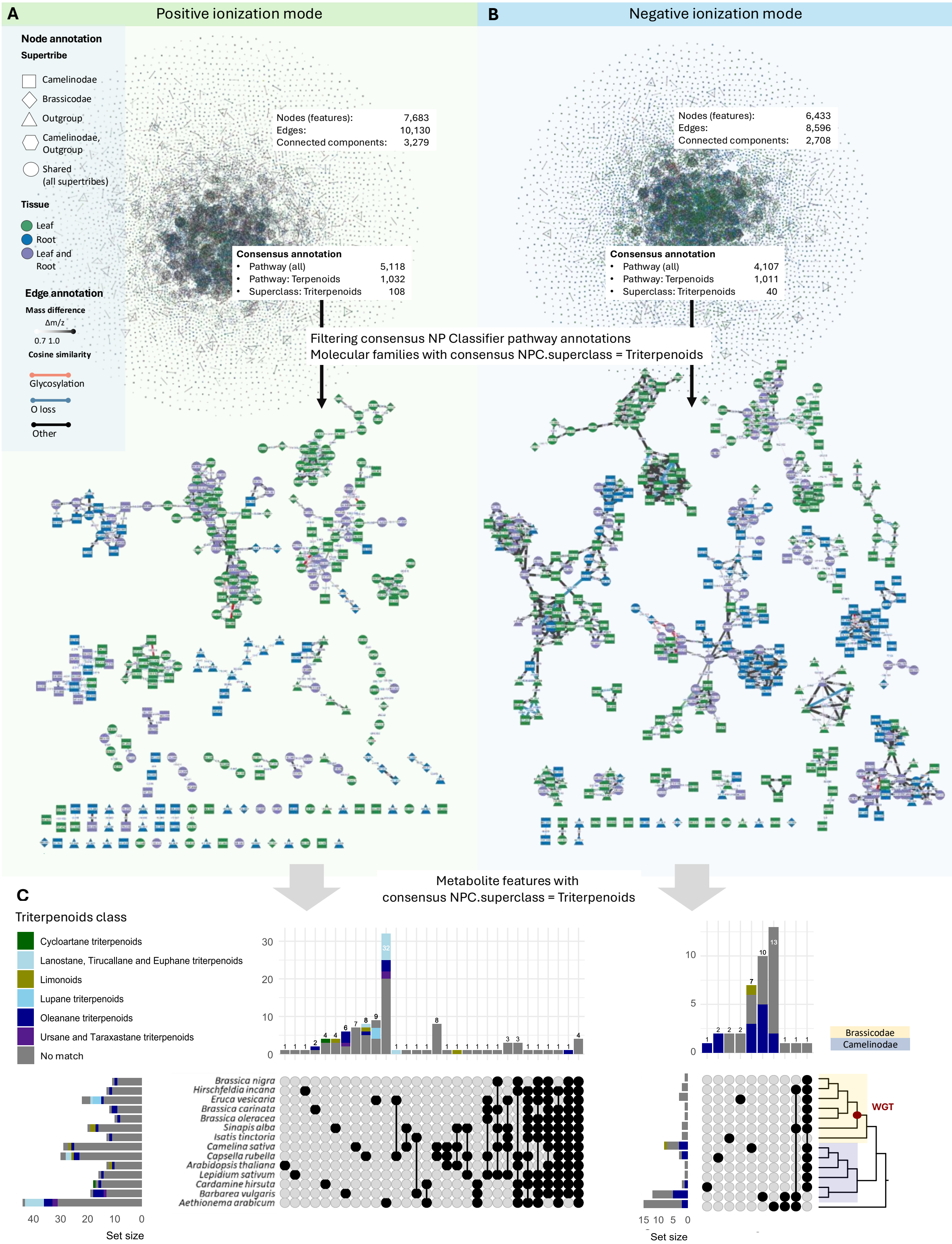

Figure S5: Distribution of triterpenoid consensus superclass annotations on the NP.Classifier superclass level. Feature-Based Molecular Networks (FBMNs) in (**a**) positive and (**b**) negative ionization mode. Node shapes represent presence/absence in supertribes, and node colors represent presence/absence in

tissue types. Edges were annotated with biotransformations corresponding to glycosylation (162.05 Da, 146.06 Da, rhamnose; glucose or galactose; 176.03 Da, glucuronide; 133.05 Da, arabinoside; 324.1 Da, double glycosylation; 309.12 Da, rhamnose and glucose) and oxidation (15.995 Da). Consensus annotations of NP.Classifier superclass "triterpenoids" were filtered in both ionization modes. **C:** Upset plots showing distribution of NP.Classifier class annotations across species for metabolite features consistently annotated as "triterpenoids" on the superclass ontology level. Triterpenoid consensus annotations without a matching class annotation across at least two annotation tools are indicated in grey ("no match"). The red dot on the phylogenetic tree indicates the Whole Genome Triplication (WGT) event in the Brassicodae clade.

The unique profiles of species in the Camelinodae supertribe are characterized by triterpenoids

Terpenoid annotations were disproportionally enriched in unique profiles of species, specifically within the Camelinodae supertribe. The largest unique profiles of annotated terpenoids were comparable in size between *C. sativa* (92, 33.5%) and *C. rubella* (77, 26%), and comprised mostly monoterpenoid annotations in *C. rubella,* which were underrepresented in *C. sativa* (Fig. S5). To further explore this pattern, we investigated compound class annotations on the superclass level to determine the distribution of mono-, di-, sesqui- and triterpenoids across species and supertribes. We found that annotated terpenoid profiles were characterized by mono-, di- and triterpenoid superclasses across supertribes, with monoterpenoids dominating in negative ionization mode.

Fewer consensus NP.Classifier superclass and class annotations were obtained for features detected in negative mode (Fig. S5b), potentially attributed to missing annotations in DreaMS v1.0 being presently restricted to positive ionization mode analysis. Triterpenoid superclass annotations had particularly inconsistent annotations on the class ontology level (Fig. S5, Material S6), potentially related to molecule size and complexity in comparison with mono- and diterpenoids.

Consistently annotated mass features within the triterpenoid superclass belong to the classes of lupane, oleanane, lanostane, tirucallane, and euphane triterpenoids in positive ionization mode (Fig. S5a), whereas only putative oleanane-type triterpenoids and a single limonoid were annotated in negative ionization mode (Fig. S5b). Characteristic fragmentation patterns in MS2 under negative ionization mode may facilitate the annotation of oleanane-type triterpenoids, biasing compound class representation. The largest proportions of unique triterpenoid annotations were found in *A. arabicum*, *B. vulgaris*, and *C. sativa*. The unique triterpenoid

profile of *B. vulgaris* consisted predominantly of putative oleanane triterpenoids in both ionization modes. In *C. sativa*, the unique triterpenoid profile comprised four consistent class annotations (putative oleanane triterpenoids and limonoids) in negative ionization mode (Fig. S5b), whilst the positive ionization mode did not result in any consistent class annotations (Fig. S5a). Thus, compound class predictions overlap with library matches obtained for previously described oleanane triterpenoid saponins in *B. vulgaris* (Khakimov *et al*., 2016; Shinoda *et al*., 2002). Furthermore, consistent annotations indicate the presence of putative oleanane triterpenoid saponins in other species, primarily in the Camelinodae supertribe.

Analysis of computational annotations under negative ionization mode reveal putative triterpenoid saponins in *Camelina sativa*, *Capsella rubella*, and *Cardamine hirsuta*

Motif_90, motif_335 and motif_288 were specifically distributed across features with matching superclass and class annotations, while motif_43, motif_21 and motif_99 were not associated with matching terpenoid consensus annotations on any chemical ontology level.

Motif_90 was extracted from a spectrum with library match to saikosaponin A (Wikidata: Q7499688) in *B. vulgaris*, a triterpenoid saponin previously characterized in a traditional Chinese medicine derived from extracts of *Bupleurum* spp. (Apiaceae; Yuan *et al*., 2017). However, the aglycone peak (m/z 455) and sugar losses were previously described by Khakimov *et al*. (2016) and Nielsen *et al.* (2010) in *B. vulgaris* G-type. Motif_90 is characterized by five mass peaks (m/z 161.04, 176.06, 205.06, 367.11, 631.39) corresponding to fragmentation patterns of triterpenoid saponin aglycones (Tsugawa *et al*., 2019), and two optimized losses (370.11 Da, 206.05 Da) shown in Figure S6.

Distribution of motif_90 was found to be limited to leaf tissue profiles of *B. vulgaris* and *Eruca vesicaria*. In *B. vulgaris*, three aglycones (m/z 469, 471, 455) in network component 854, and corresponding sugar losses were tentatively identified as described in the literature (Khakimov *et al*., 2016; Shinoda *et al*., 2002; Fig. S6a). Motif_335 is shared by eight metabolite features with probability scores ^{3}0.5 and overlap scores ^{3}0.15, six of which were annotated as terpenoids by NP.Classifier (Material S6). While motif_355 is widely distributed across species in Camelinodae and Brassicodae, motif_90 is restricted to *B. vulgaris* and *E. vesicaria*. However, mass peaks (m/z 101.02 and 161.05) in matching library spectra (feature ID: 6663) were absent in motif_90, but present in motif_335 (Fig. S6b). In addition, most optimized fragment peaks of motif_335 (m/z 101.02, 113.02, 161.05, 221.07) overlap with motif_288 (Material S3). Motif_335 and motif_288 were found in leaf and root tissue across species of Camelinodae

and Brassicodae supertribes, as well as in the outgroup. Five metabolite features (probability score ³0.5, overlap score ³0.2) share motif_288, four of which have a consensus annotation in the terpenoid pathway, while two share a triterpenoid superclass consensus annotation (Material S6).

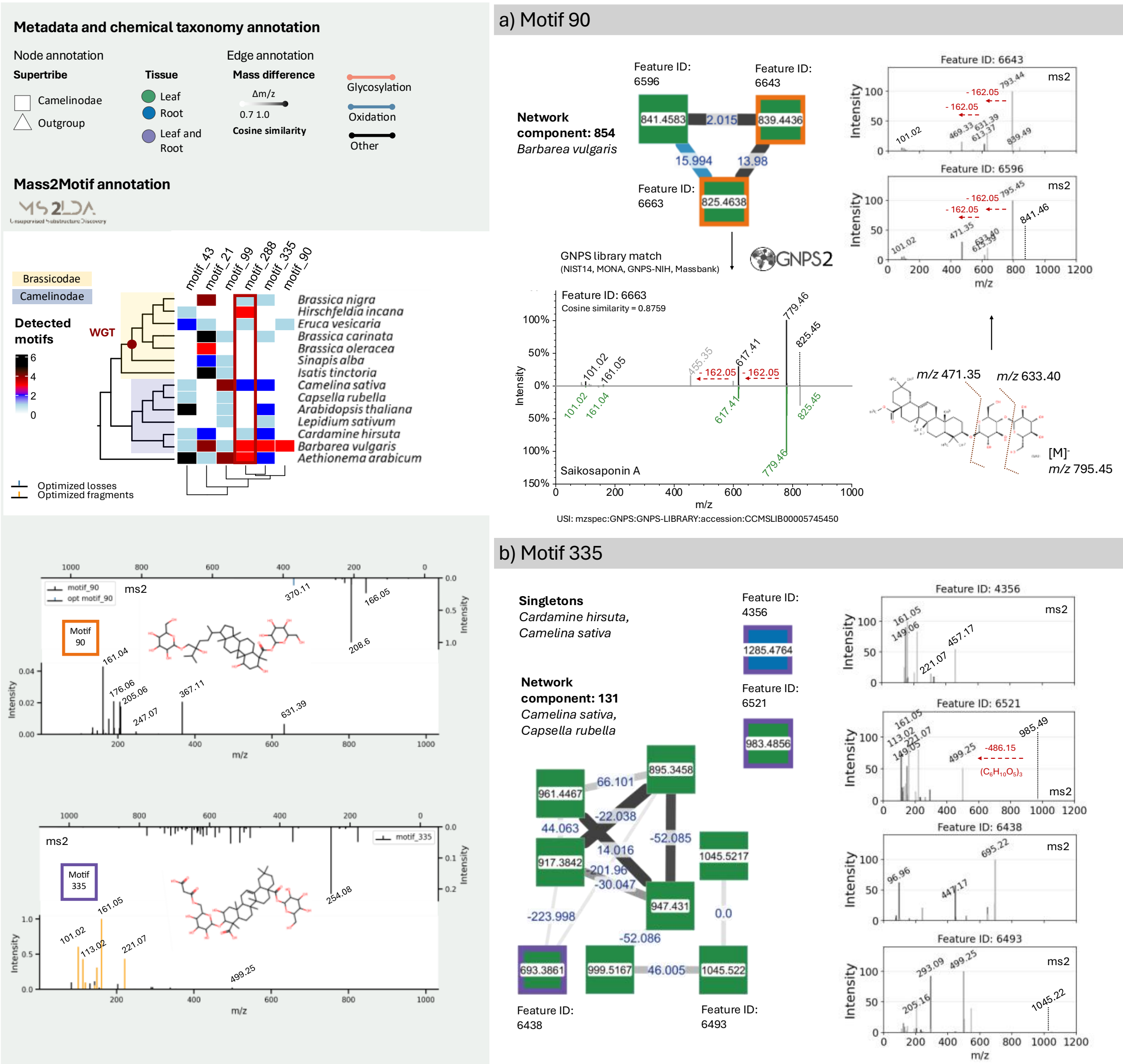

Figure S6. Mappings of Mass2Motifs extracted with MS2LDA in negative ionization mode. Mass2Motifs associated with fragmentation patterns and annotated substructures of oleanane-type triterpenoids were mapped on molecular families in the Feature-Based Molecular Network (FBMN). Node shape and color represent presence/absence in supertribes and tissues, respectively. Edges were annotated with biotransformations corresponding to glycosylation (162.05 Da, glucose) and oxidation (15.995 Da). **A:** Distribution of motif_90 across a selection of features, and GNPS library match in *Barbarea vulgaris*. **B:** Distribution of motif_288 on a selection of features in Camelinodae species.

Chemical taxonomy across 17 species reflects distinct hierarchical clustering patterns on chemical ontology levels when comparing leaf and root tissue

The total number of unique annotations in root tissue (n = 233, 26.0%) was comparable with the number of features annotated in leaf tissue (188, 21.0%). Additionally, the largest proportion of annotated metabolite features is shared across tissues between Camelinodae and Brassicodae supertribes (177, 21.1%, Fig. 6a and S7b). Hierarchical complete clustering of the resulting set of 895 features was followed by the construction of tanglegrams for comparison with species phylogeny according to Hendriks *et al*. (2024) using mass features from both leaf and root tissues (Fig. 7b). Furthermore, hierarchical complete clustering and resulting tanglegrams were compared for the total number of features extracted from leaf (n = 662) (Fig. 6c) and root tissue (n = 707) (Fig. 8d), including features extracted from both tissue types (n = 474). The numbers of annotated metabolite features extracted exclusively from leaf (188) and root (233) tissue are given in Table 2 and in the Venn diagram shown in Fig. S7b.

Table 2: Number of metabolite features obtained for integrated batches including LC-MS/MS data of leaf and root extracts of 17 species

**Unfiltered consensus spectra**	11,271 in positive mode<br>14,329 in negative mode
**InChI with confidence exact score >0.6 obtained in SIRIUS v.6.1.0**	1,048
**Unique InChI in consensus spectra**	
Leaf-specific profile	188
**Leaf profile (total)**	188 + 474 = 662
Root-specific profile	233
**Root profile (total)**	233 + 474 = 707
Shared profile (leaf/root)	474
**Total (all features)**	895

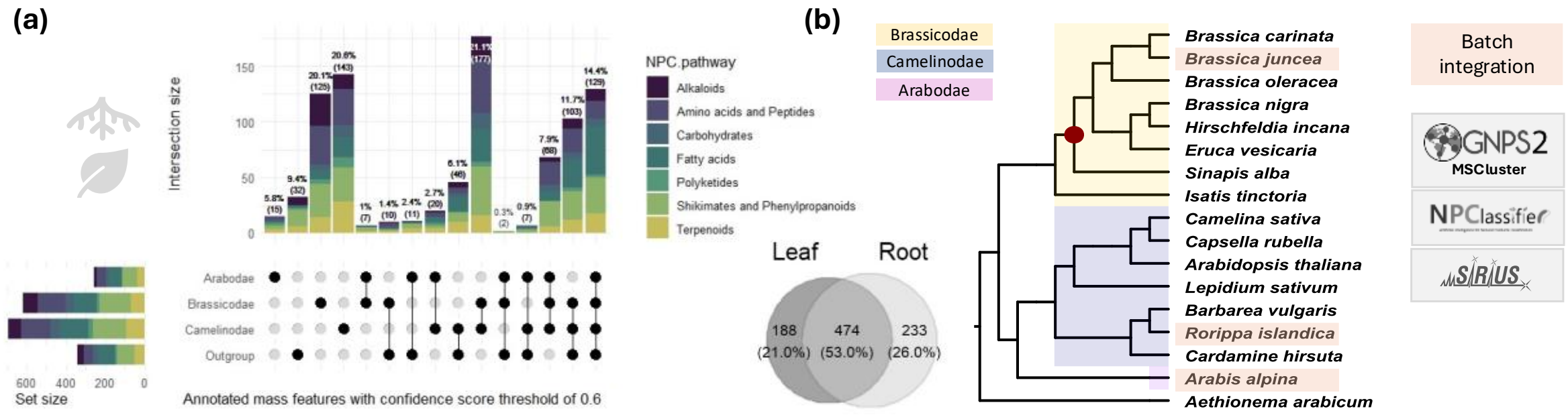

Figure S7: Structural annotations in 17 species across leaf and root tissue and across two batches of LC-MS/MS data. Untargeted LC-MS/MS data was aligned by applying the MS-Cluster algorithm on GNPS2, and subsequently filtering for structural annotations obtained in SIRIUS with a Confidence Exact Score threshold of 0.6. Multiple identical structural annotations potentially resulting from in-source fragmentation and adduct formation were grouped. (a) , Upset plot showing the distribution across NP.Classifier "pathway" annotations of structural predictions by SIRIUS v6.1.0. (b) Phylogenetic tree of species included in hierarchical clustering analysis. WGT, Whole-Genome Triplication event indicated by red circle on the phylogenetic tree. Venn diagram of shared and unique annotations in leaf and root tissue after filtering.

Hierarchical clustering of Brassica species reflects relationships of shared sub-genomes in descendants of *B. nigra* and *B. oleracea*

To investigate the metabolic distance among species within the *Brassica* genus, we included *B. juncea*, which harbors the A and B genomes derived from its progenitor species *B. rapa* and *B. nigra* (*Mutarda nigra*). In leaf tissue-specific hierarchical clustering in the metabolomic tree, diploid *B. nigra* and *B. oleracea* group with the polyploids *B. juncea* (an AB genome species) and *B. carinata* (a BC genome species), respectively (Fig. 6c). All included *Brassica* species formed a distinct, monophyletic clade based on leaf tissue profiles, indicating conserved tissue-specific chemistry within this genus. In contrast, *B. nigra* and *B. juncea* harboring the B genome were split from *B. oleracea* and *B. carinata* harboring the C genome in root tissue-specific hierarchical clustering (Fig. 6d). Interestingly, the B-genome species *B. nigra* groups with its close relative *H. incana* when only including root-specific profiles. However, when leaf and root tissue profiles are jointly analyzed, *H. incana* clusters with the C-genome species *B. oleracea* in a separate cluster within the metabolomic tree, while *B. nigra*, *B. juncea* and *B. carinata* form a monophyletic clade (Fig. 6b). Consistent grouping of *B. nigra* and *B. juncea* suggests that the metabolite profile of *B. juncea* is consistently characterized by the shared B

genome across leaf and root tissue. In contrast, *B. carinata* grouping consistently with *B. oleracea* suggests that the metabolite profile in *B. carinata* is more determined by the C genome rather than the B genome, particularly in root tissue.

We included merely a filtered set of metabolite features in our analysis, restricted to high-confidence structure predictions in SIRIUS. However, hierarchical clustering of *B. nigra*, *B. juncea*, *B. carinata*, and *B. oleracea* in the metabolomic tree is observed across all hierarchical clustering scenarios, including unfiltered aligned metabolite features (Figure S8). The inclusion of *B. rapa* and *B. napus* covering A and AC genomes in metabolic fingerprinting for chemical taxonomy may provide further insights into the effects of sub-genome dominance of A versus B and C genomes in future research.

(a)

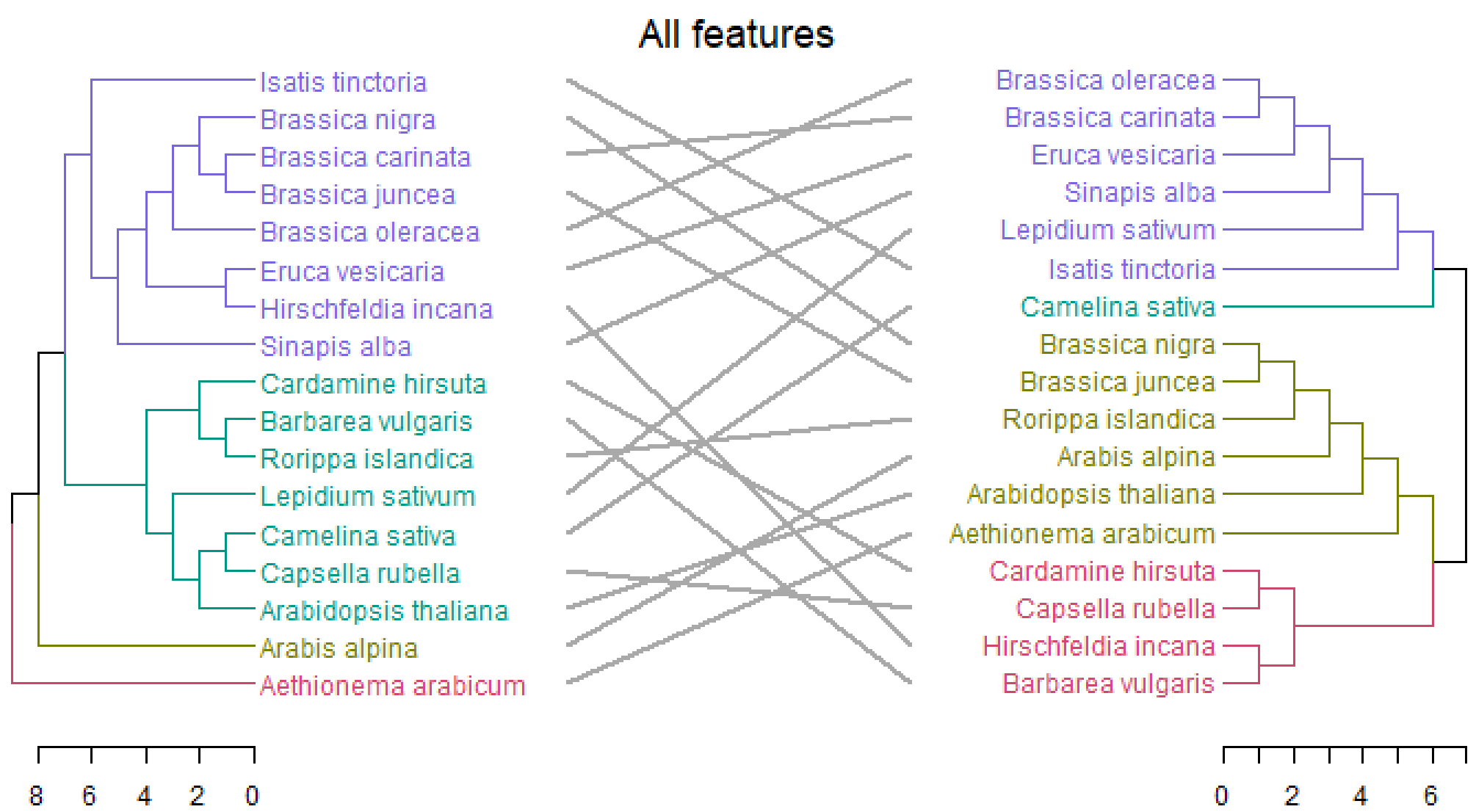

(b)

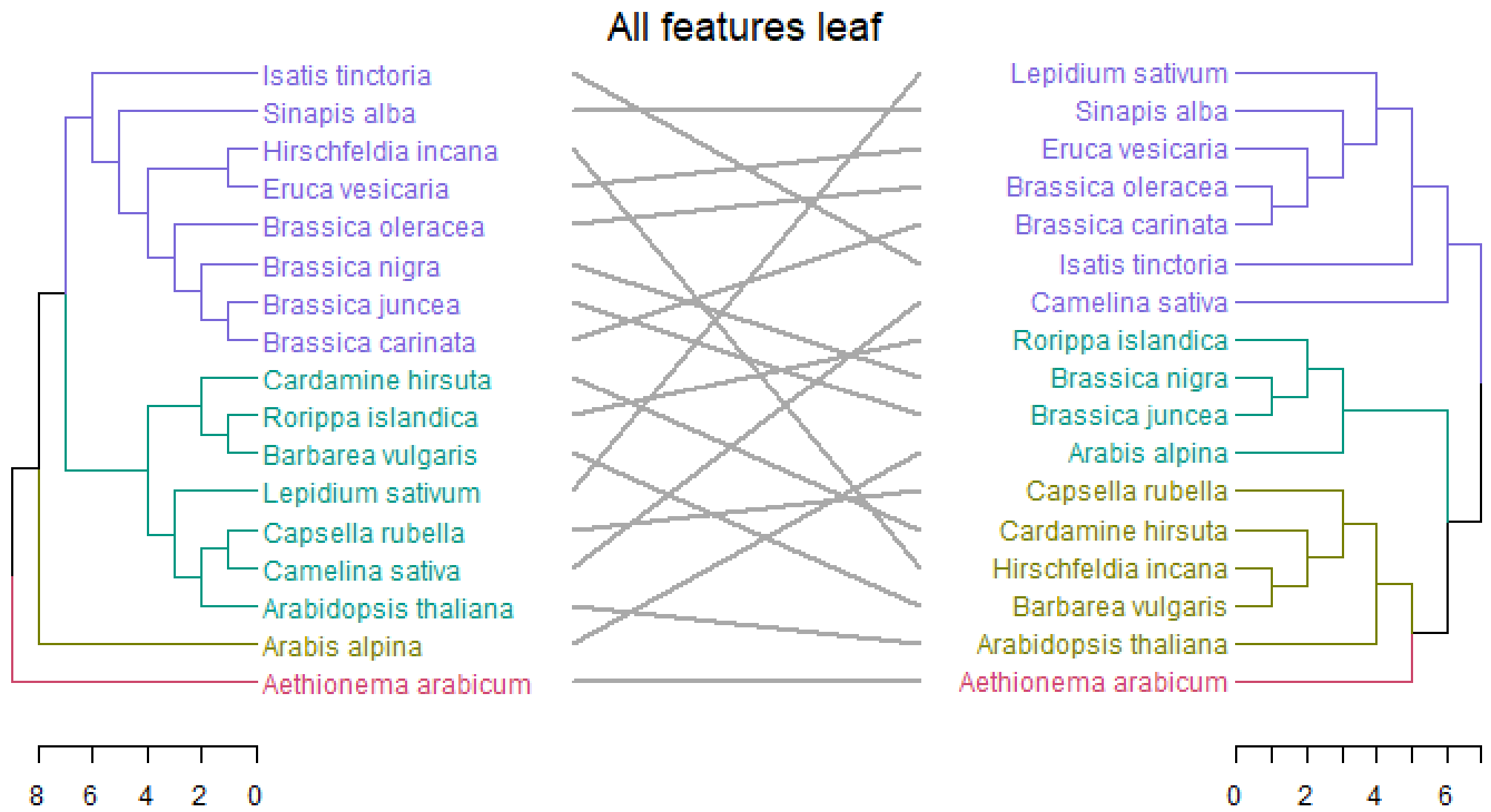
All features leaf
Isatis tinctoria
Sinapis alba
Hirschfeldia incana
Eruca vesicaria
Brassica oleracea
Brassica nigra
Brassica juncea
Brassica carinata
Cardamine hirsuta
Rorippa islandica
Barbarea vulgaris
Lepidium sativum
Capsella rubella
Camelina sativa
Arabidopsis thaliana
Arabis alpina
Aethionema arabicum
8 6 4 2 0
Lepidium sativum
Sinapis alba
Eruca vesicaria
Brassica oleracea
Brassica carinata
Isatis tinctoria
Camelina sativa
Rorippa islandica
Brassica nigra
Brassica juncea
Arabis alpina
Capsella rubella
Cardamine hirsuta
Hirschfeldia incana
Barbarea vulgaris
Arabidopsis thaliana
Aethionema arabicum
0 2 4 6

(c)

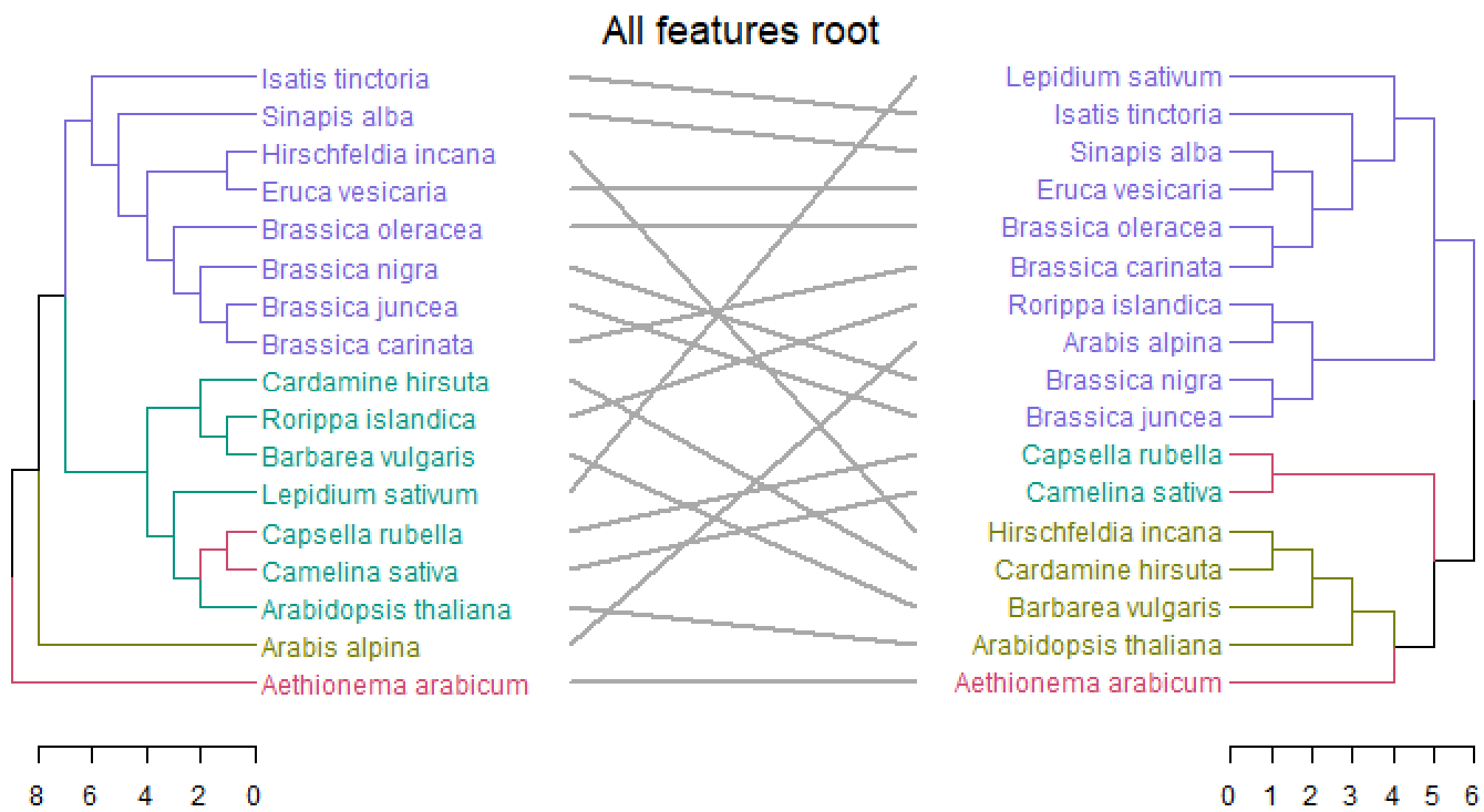
All features root
Isatis tinctoria
Sinapis alba
Hirschfeldia incana
Eruca vesicaria
Brassica oleracea
Brassica nigra
Brassica juncea
Brassica carinata
Cardamine hirsuta
Rorippa islandica
Barbarea vulgaris
Lepidium sativum
Capsella rubella
Camelina sativa
Arabidopsis thaliana
Arabis alpina
Aethionema arabicum
8 6 4 2 0
Lepidium sativum
Isatis tinctoria
Sinapis alba
Eruca vesicaria
Brassica oleracea
Brassica carinata
Rorippa islandica
Arabis alpina
Brassica nigra
Brassica juncea
Capsella rubella
Camelina sativa
Hirschfeldia incana
Cardamine hirsuta
Barbarea vulgaris
Arabidopsis thaliana
Aethionema arabicum
0 1 2 3 4 5 6

(d)

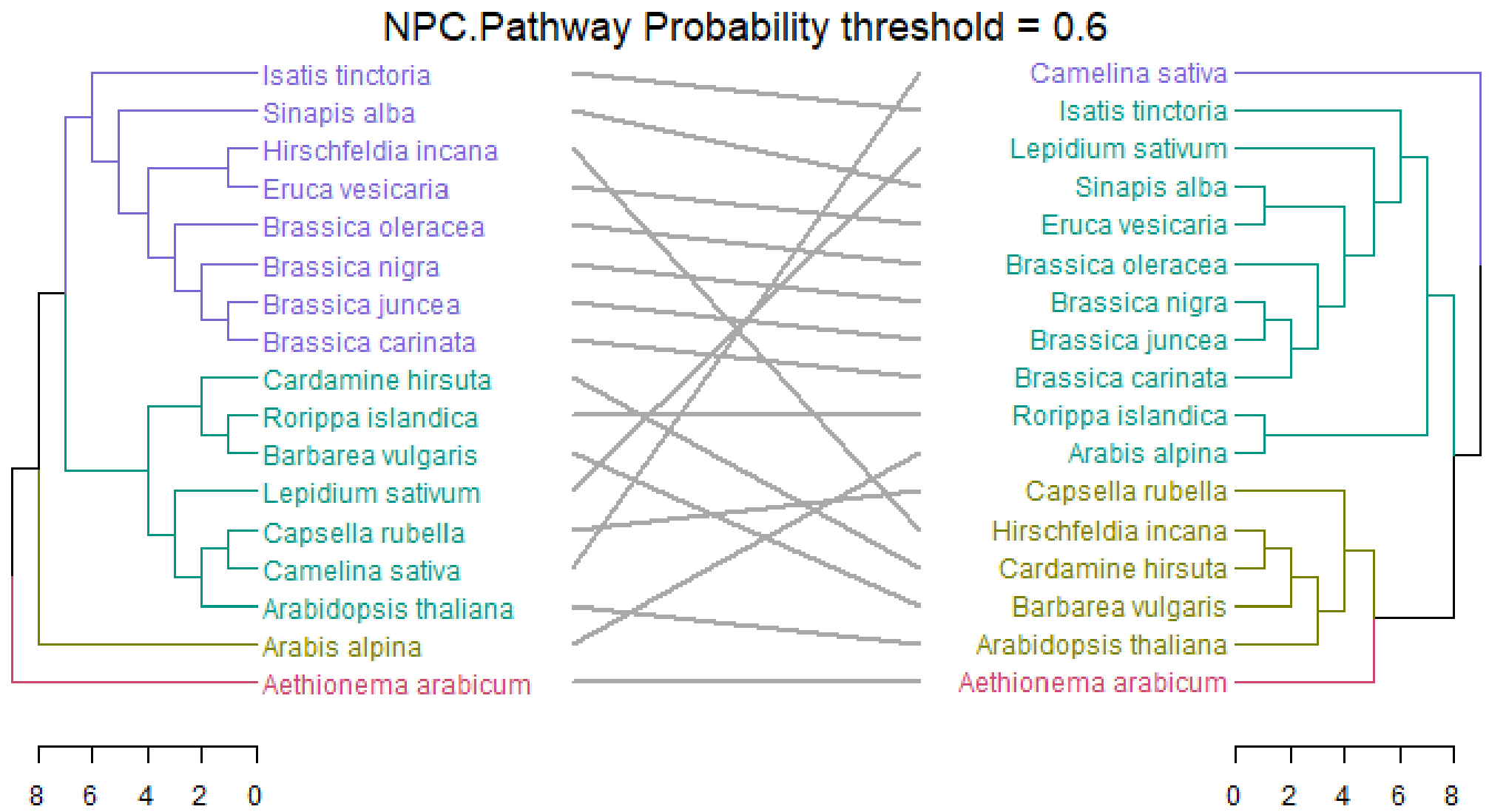
NPC.Pathway Probability threshold = 0.6
Isatis tinctoria
Sinapis alba
Hirschfeldia incana
Eruca vesicaria
Brassica oleracea
Brassica nigra
Brassica juncea
Brassica carinata
Cardamine hirsuta
Rorippa islandica
Barbarea vulgaris
Lepidium sativum
Capsella rubella
Camelina sativa
Arabidopsis thaliana
Arabis alpina
Aethionema arabicum
8 6 4 2 0
Camelina sativa
Isatis tinctoria
Lepidium sativum
Sinapis alba
Eruca vesicaria
Brassica oleracea
Brassica nigra
Brassica juncea
Brassica carinata
Rorippa islandica
Arabis alpina
Capsella rubella
Hirschfeldia incana
Cardamine hirsuta
Barbarea vulgaris
Arabidopsis thaliana
Aethionema arabicum
0 2 4 6 8

(e)

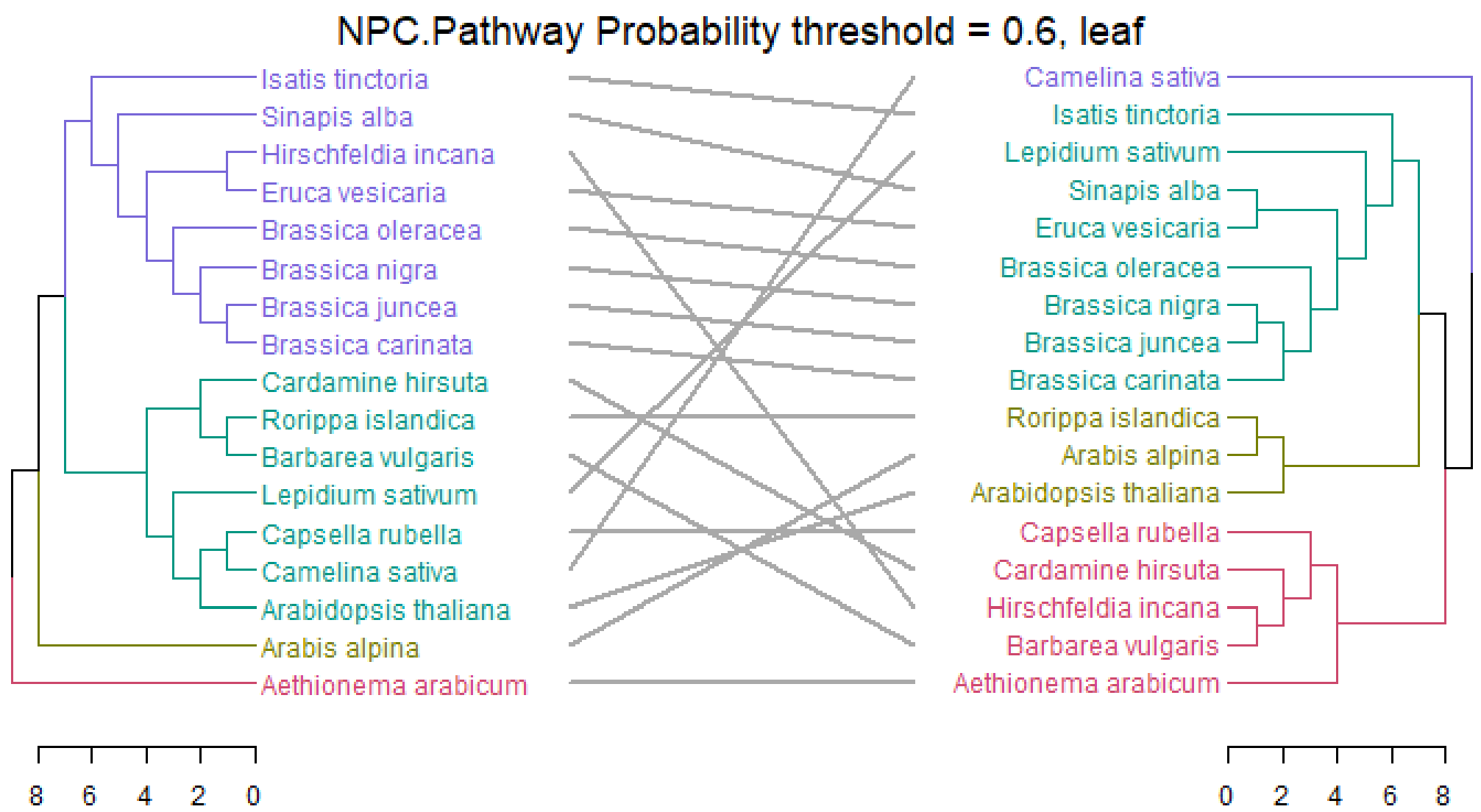
NPC.Pathway Probability threshold = 0.6, leaf
Isatis tinctoria
Sinapis alba
Hirschfeldia incana
Eruca vesicaria
Brassica oleracea
Brassica nigra
Brassica juncea
Brassica carinata
Cardamine hirsuta
Rorippa islandica
Barbarea vulgaris
Lepidium sativum
Capsella rubella
Camelina sativa
Arabidopsis thaliana
Arabis alpina
Aethionema arabicum
8 6 4 2 0
Camelina sativa
Isatis tinctoria
Lepidium sativum
Sinapis alba
Eruca vesicaria
Brassica oleracea
Brassica nigra
Brassica juncea
Brassica carinata
Rorippa islandica
Arabis alpina
Arabidopsis thaliana
Capsella rubella
Cardamine hirsuta
Hirschfeldia incana
Barbarea vulgaris
Aethionema arabicum
0 2 4 6 8

(f)

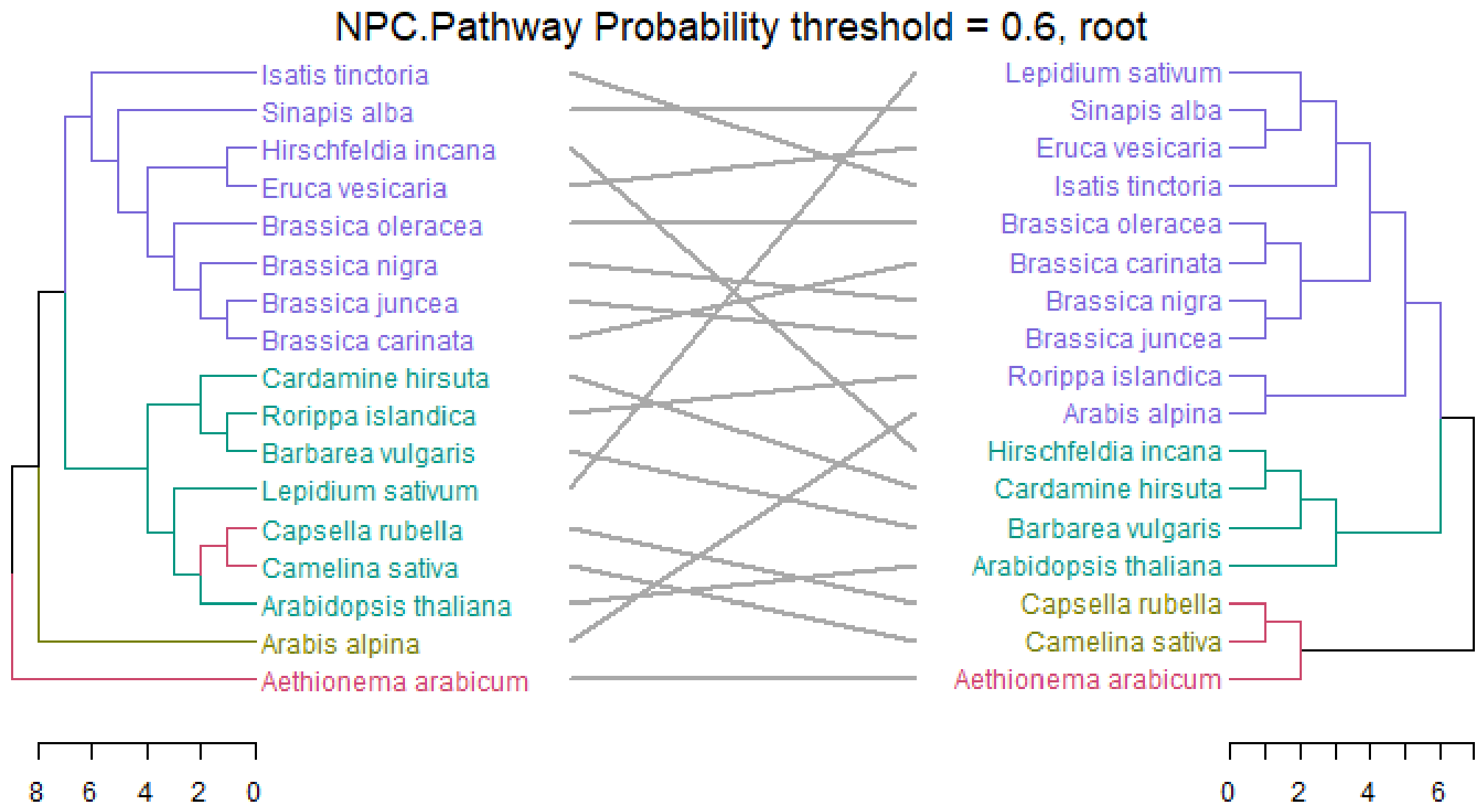
NPC.Pathway Probability threshold = 0.6, root
Isatis tinctoria
Sinapis alba
Hirschfeldia incana
Eruca vesicaria
Brassica oleracea
Brassica nigra
Brassica juncea
Brassica carinata
Cardamine hirsuta
Rorippa islandica
Barbarea vulgaris
Lepidium sativum
Capsella rubella
Camelina sativa
Arabidopsis thaliana
Arabis alpina
Aethionema arabicum
Lepidium sativum
Sinapis alba
Eruca vesicaria
Isatis tinctoria
Brassica oleracea
Brassica carinata
Brassica nigra
Brassica juncea
Rorippa islandica
Arabis alpina
Hirschfeldia incana
Cardamine hirsuta
Barbarea vulgaris
Arabidopsis thaliana
Capsella rubella
Camelina sativa
Aethionema arabicum
8 6 4 2 0
0 2 4 6

(g)

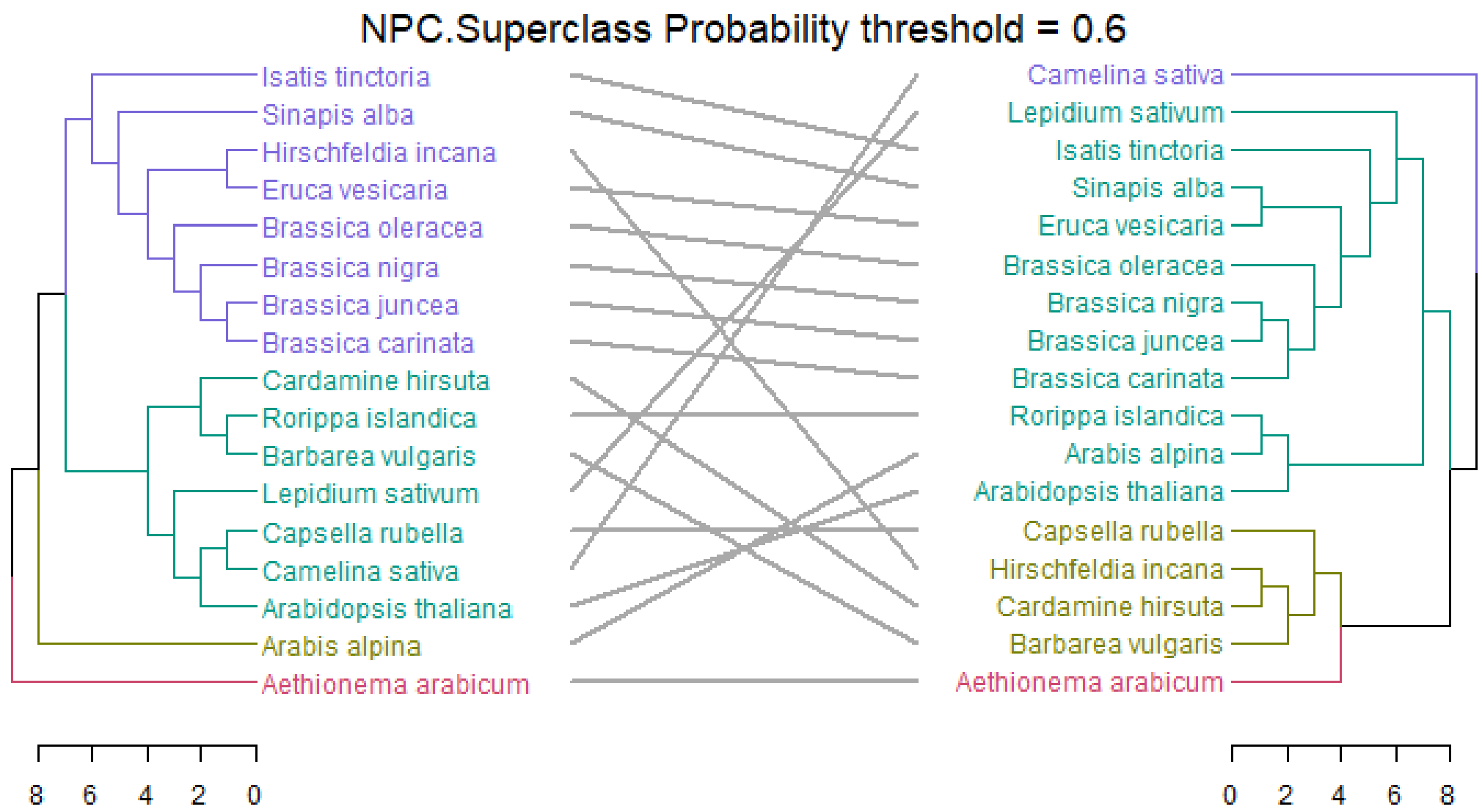
NPC.Superclass Probability threshold = 0.6
Isatis tinctoria
Sinapis alba
Hirschfeldia incana
Eruca vesicaria
Brassica oleracea
Brassica nigra
Brassica juncea
Brassica carinata
Cardamine hirsuta
Rorippa islandica
Barbarea vulgaris
Lepidium sativum
Capsella rubella
Camelina sativa
Arabidopsis thaliana
Arabis alpina
Aethionema arabicum
Camelina sativa
Lepidium sativum
Isatis tinctoria
Sinapis alba
Eruca vesicaria
Brassica oleracea
Brassica nigra
Brassica juncea
Brassica carinata
Rorippa islandica
Arabis alpina
Arabidopsis thaliana
Capsella rubella
Hirschfeldia incana
Cardamine hirsuta
Barbarea vulgaris
Aethionema arabicum
8 6 4 2 0
0 2 4 6 8

(h)

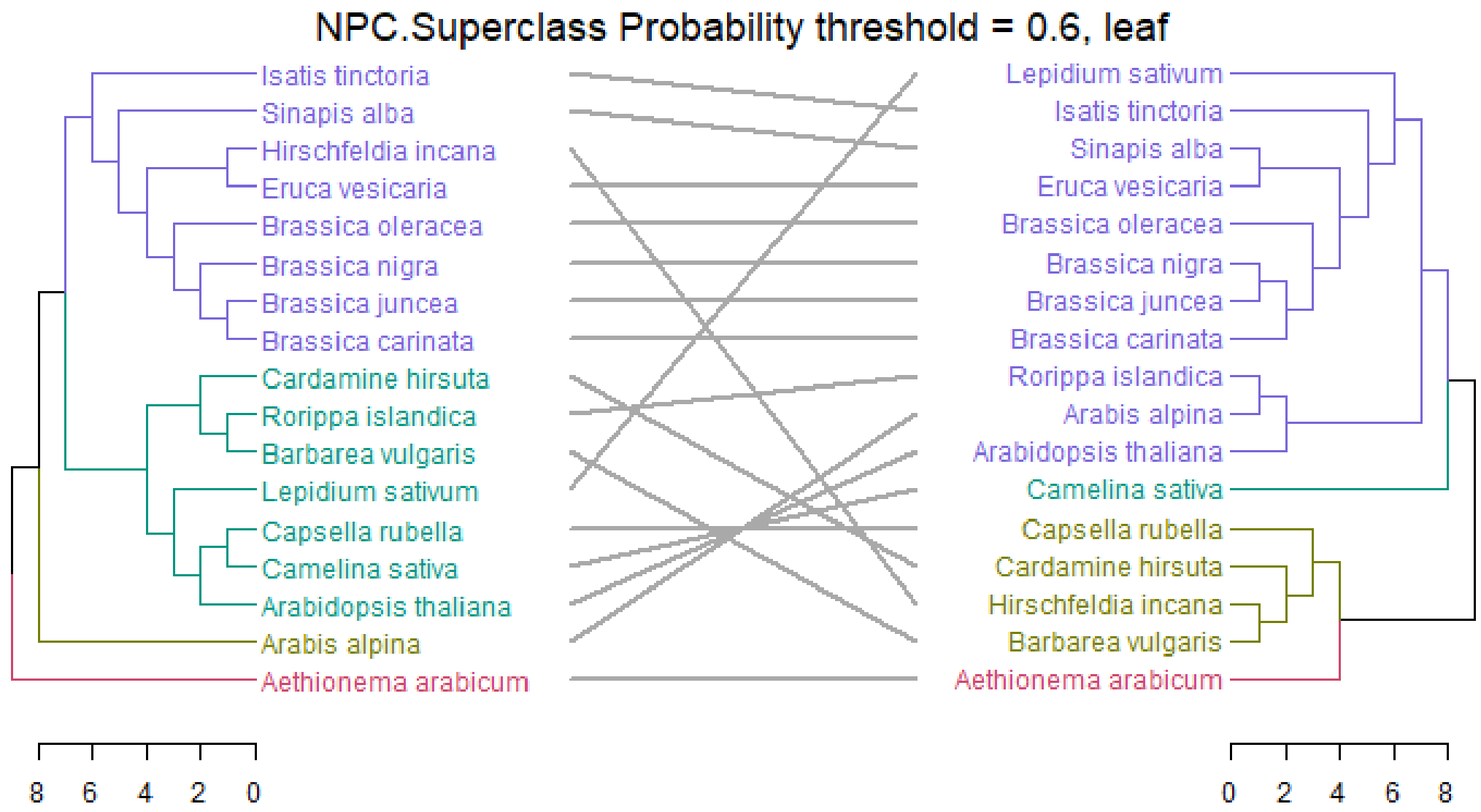
NPC.Superclass Probability threshold = 0.6, leaf
Isatis tinctoria
Sinapis alba
Hirschfeldia incana
Eruca vesicaria
Brassica oleracea
Brassica nigra
Brassica juncea
Brassica carinata
Cardamine hirsuta
Rorippa islandica
Barbarea vulgaris
Lepidium sativum
Capsella rubella
Camelina sativa
Arabidopsis thaliana
Arabis alpina
Aethionema arabicum
Lepidium sativum
Isatis tinctoria
Sinapis alba
Eruca vesicaria
Brassica oleracea
Brassica nigra
Brassica juncea
Brassica carinata
Rorippa islandica
Arabis alpina
Arabidopsis thaliana
Camelina sativa
Capsella rubella
Cardamine hirsuta
Hirschfeldia incana
Barbarea vulgaris
Aethionema arabicum
8 6 4 2 0
0 2 4 6 8

(i)

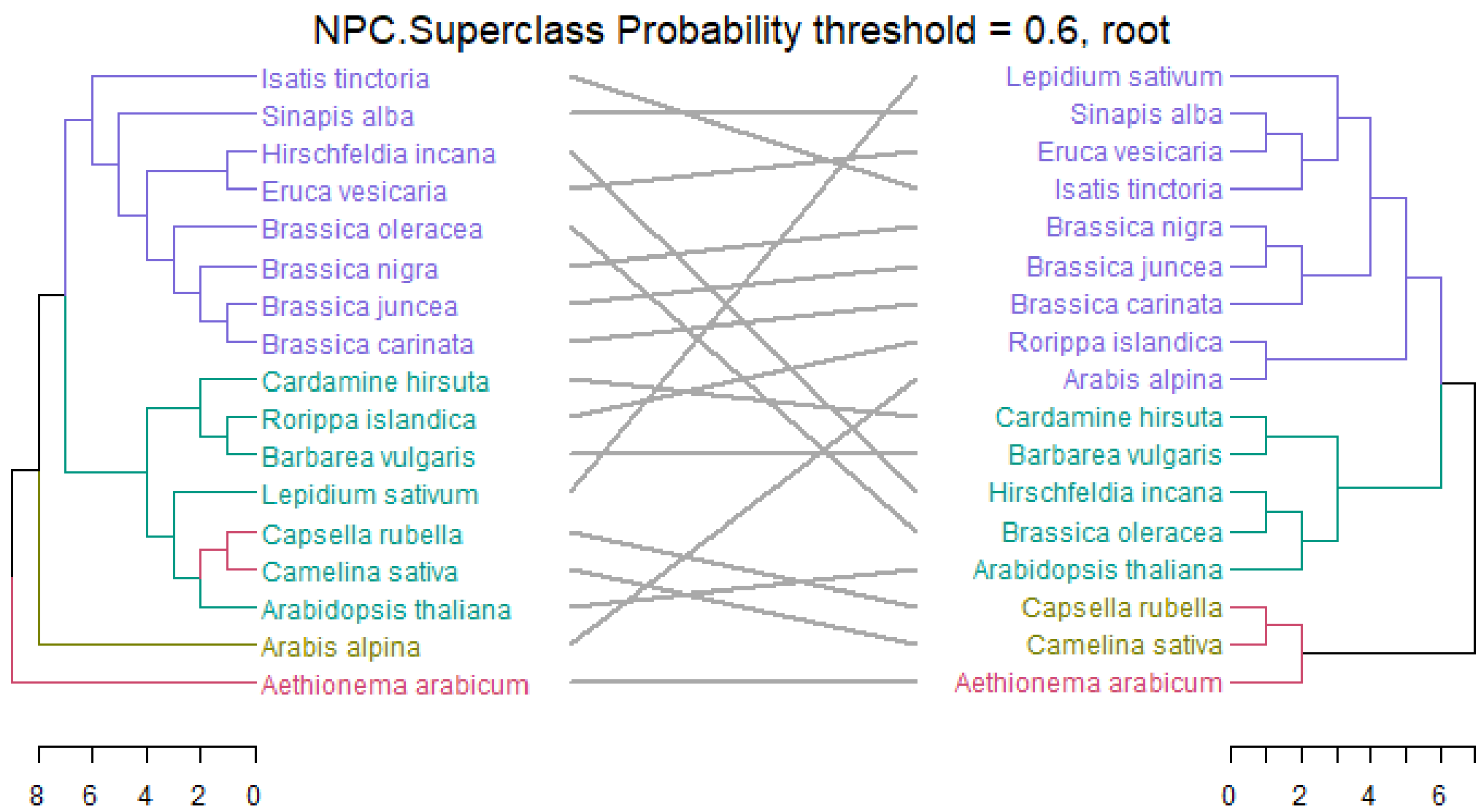
NPC.Superclass Probability threshold = 0.6, root
Isatis tinctoria
Sinapis alba
Hirschfeldia incana
Eruca vesicaria
Brassica oleracea
Brassica nigra
Brassica juncea
Brassica carinata
Cardamine hirsuta
Rorippa islandica
Barbarea vulgaris
Lepidium sativum
Capsella rubella
Camelina sativa
Arabidopsis thaliana
Arabis alpina
Aethionema arabicum
Lepidium sativum
Sinapis alba
Eruca vesicaria
Isatis tinctoria
Brassica nigra
Brassica juncea
Brassica carinata
Rorippa islandica
Arabis alpina
Cardamine hirsuta
Barbarea vulgaris
Hirschfeldia incana
Brassica oleracea
Arabidopsis thaliana
Capsella rubella
Camelina sativa
Aethionema arabicum
8 6 4 2 0
0 2 4 6

(j)

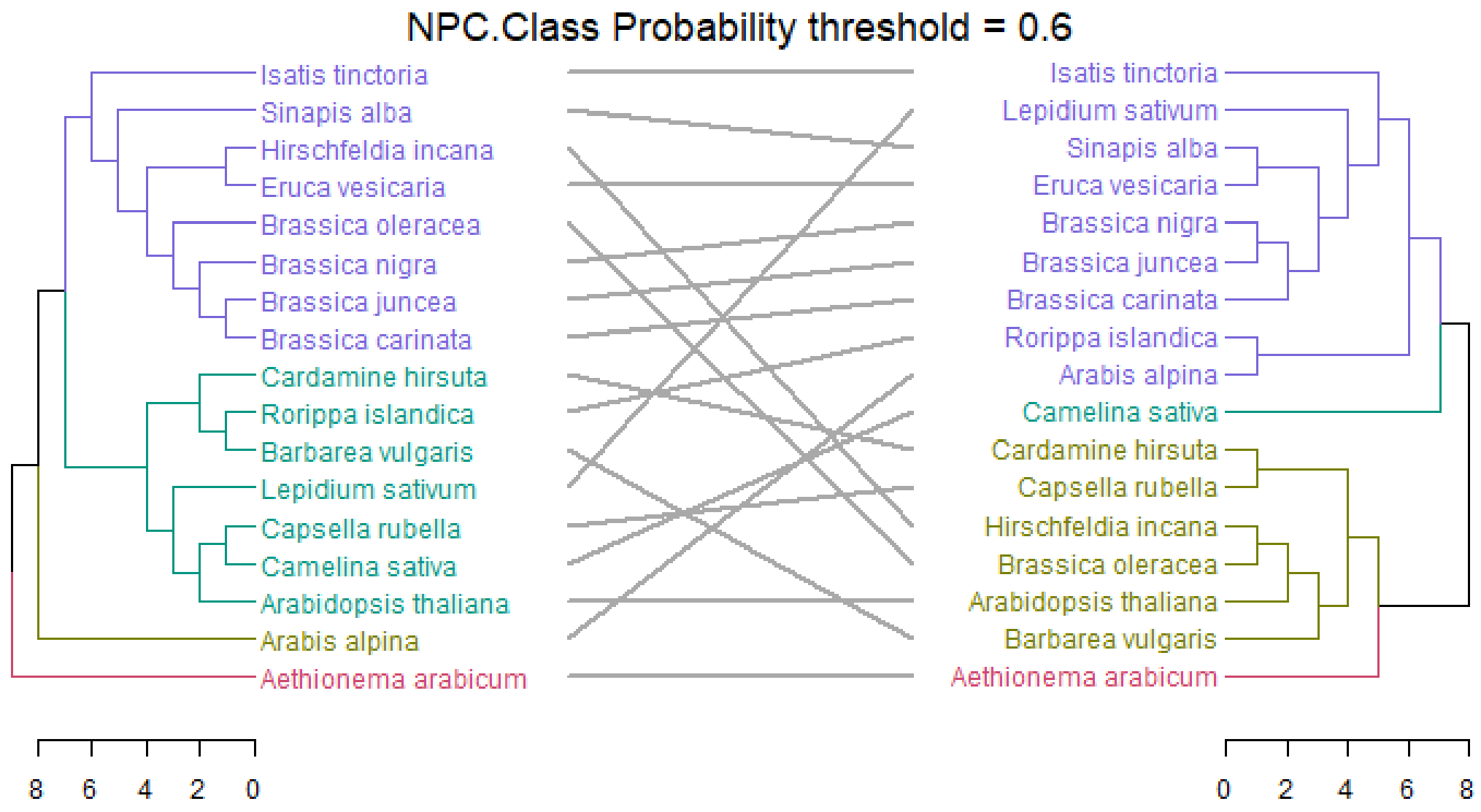
NPC.Class Probability threshold = 0.6
Isatis tinctoria
Sinapis alba
Hirschfeldia incana
Eruca vesicaria
Brassica oleracea
Brassica nigra
Brassica juncea
Brassica carinata
Cardamine hirsuta
Rorippa islandica
Barbarea vulgaris
Lepidium sativum
Capsella rubella
Camelina sativa
Arabidopsis thaliana
Arabis alpina
Aethionema arabicum
Isatis tinctoria
Lepidium sativum
Sinapis alba
Eruca vesicaria
Brassica nigra
Brassica juncea
Brassica carinata
Rorippa islandica
Arabis alpina
Camelina sativa
Cardamine hirsuta
Capsella rubella
Hirschfeldia incana
Brassica oleracea
Arabidopsis thaliana
Barbarea vulgaris
Aethionema arabicum
8 6 4 2 0
0 2 4 6 8

(k)

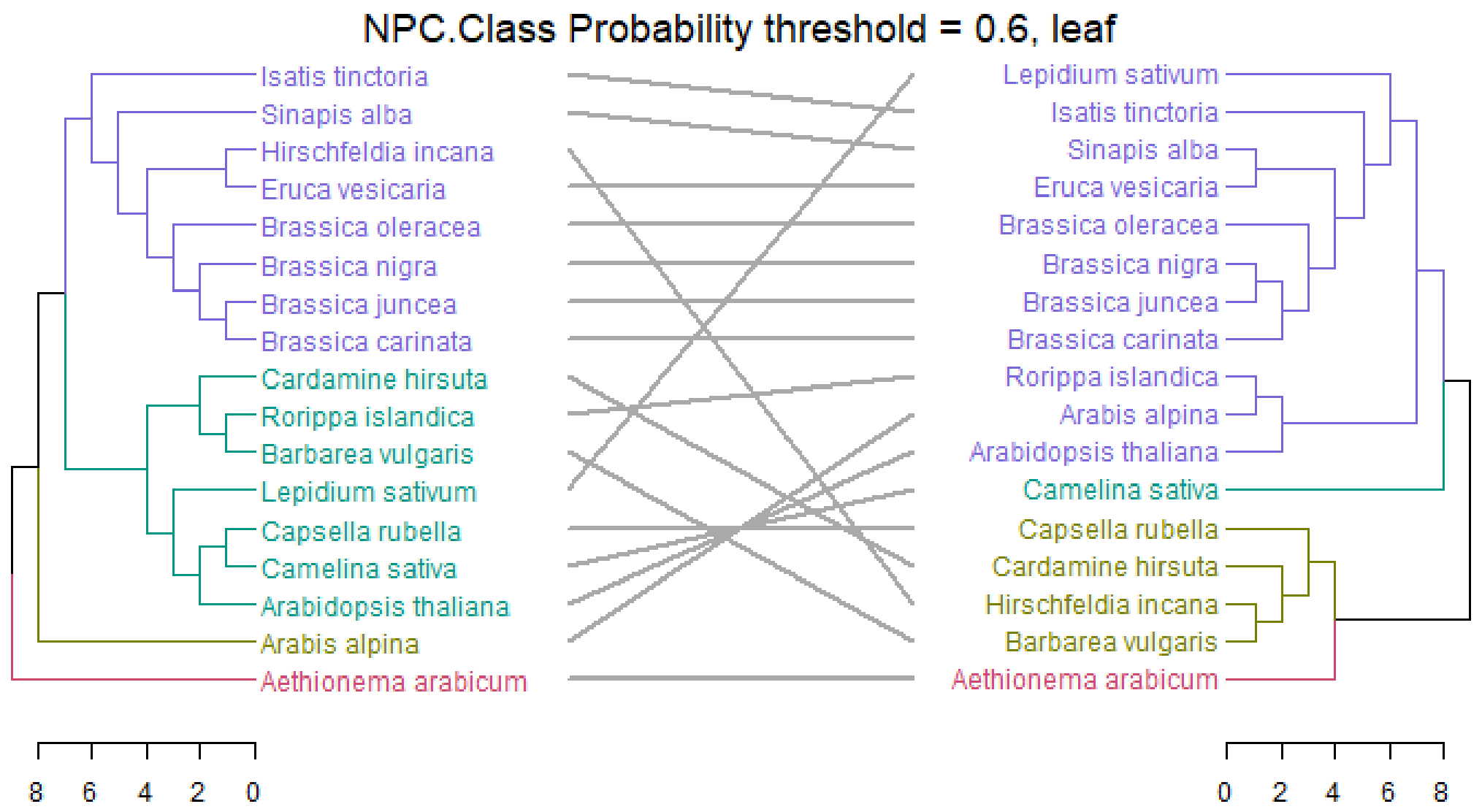
NPC.Class Probability threshold = 0.6, leaf
Isatis tinctoria
Sinapis alba
Hirschfeldia incana
Eruca vesicaria
Brassica oleracea
Brassica nigra
Brassica juncea
Brassica carinata
Cardamine hirsuta
Rorippa islandica
Barbarea vulgaris
Lepidium sativum
Capsella rubella
Camelina sativa
Arabidopsis thaliana
Arabis alpina
Aethionema arabicum
Lepidium sativum
Isatis tinctoria
Sinapis alba
Eruca vesicaria
Brassica oleracea
Brassica nigra
Brassica juncea
Brassica carinata
Rorippa islandica
Arabis alpina
Arabidopsis thaliana
Camelina sativa
Capsella rubella
Cardamine hirsuta
Hirschfeldia incana
Barbarea vulgaris
Aethionema arabicum
8 6 4 2 0
0 2 4 6 8

(l)

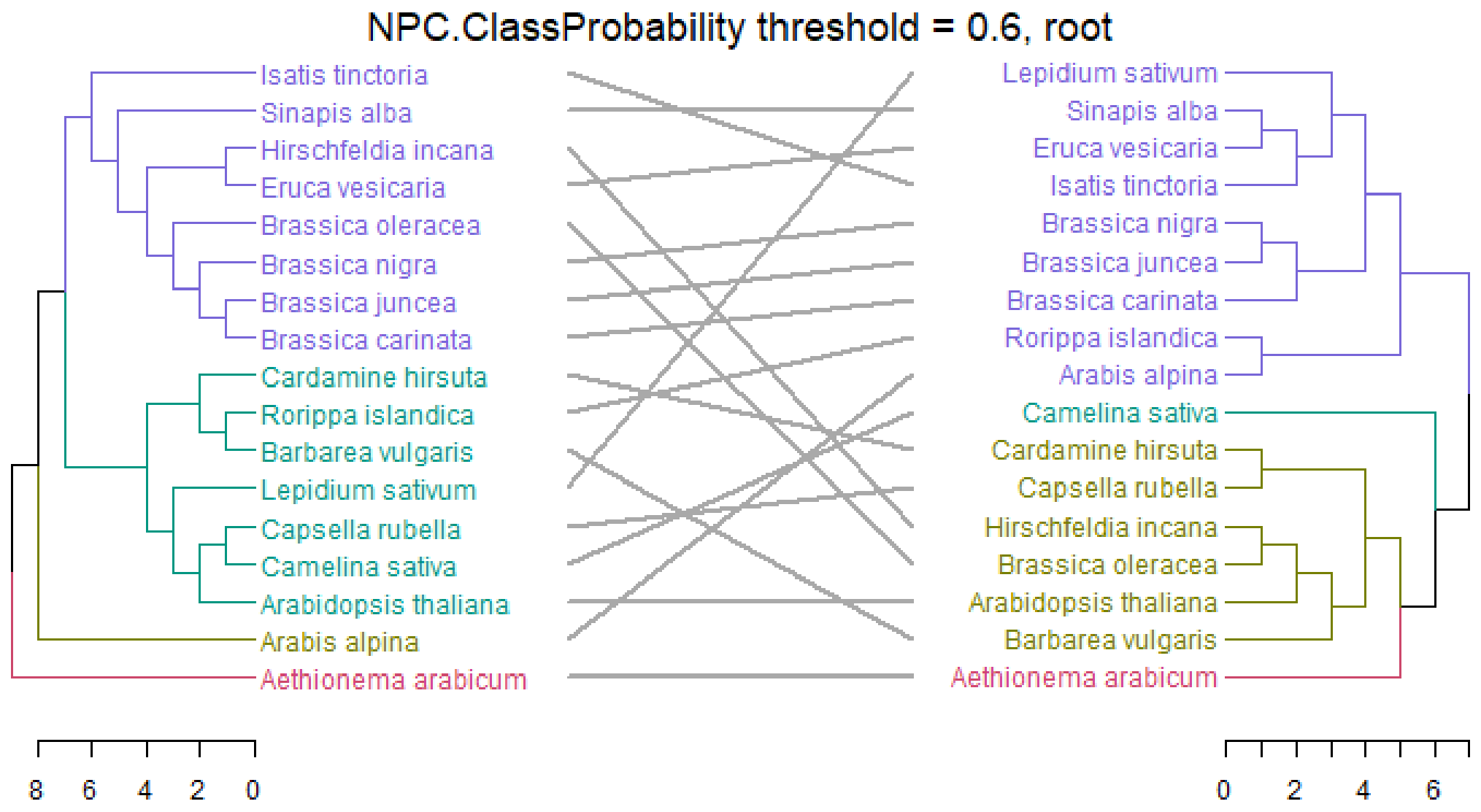
NPC.ClassProbability threshold = 0.6, root
Isatis tinctoria
Sinapis alba
Hirschfeldia incana
Eruca vesicaria
Brassica oleracea
Brassica nigra
Brassica juncea
Brassica carinata
Cardamine hirsuta
Rorippa islandica
Barbarea vulgaris
Lepidium sativum
Capsella rubella
Camelina sativa
Arabidopsis thaliana
Arabis alpina
Aethionema arabicum
Lepidium sativum
Sinapis alba
Eruca vesicaria
Isatis tinctoria
Brassica nigra
Brassica juncea
Brassica carinata
Rorippa islandica
Arabis alpina
Camelina sativa
Cardamine hirsuta
Capsella rubella
Hirschfeldia incana
Brassica oleracea
Arabidopsis thaliana
Barbarea vulgaris
Aethionema arabicum
8 6 4 2 0
0 2 4 6

(m)

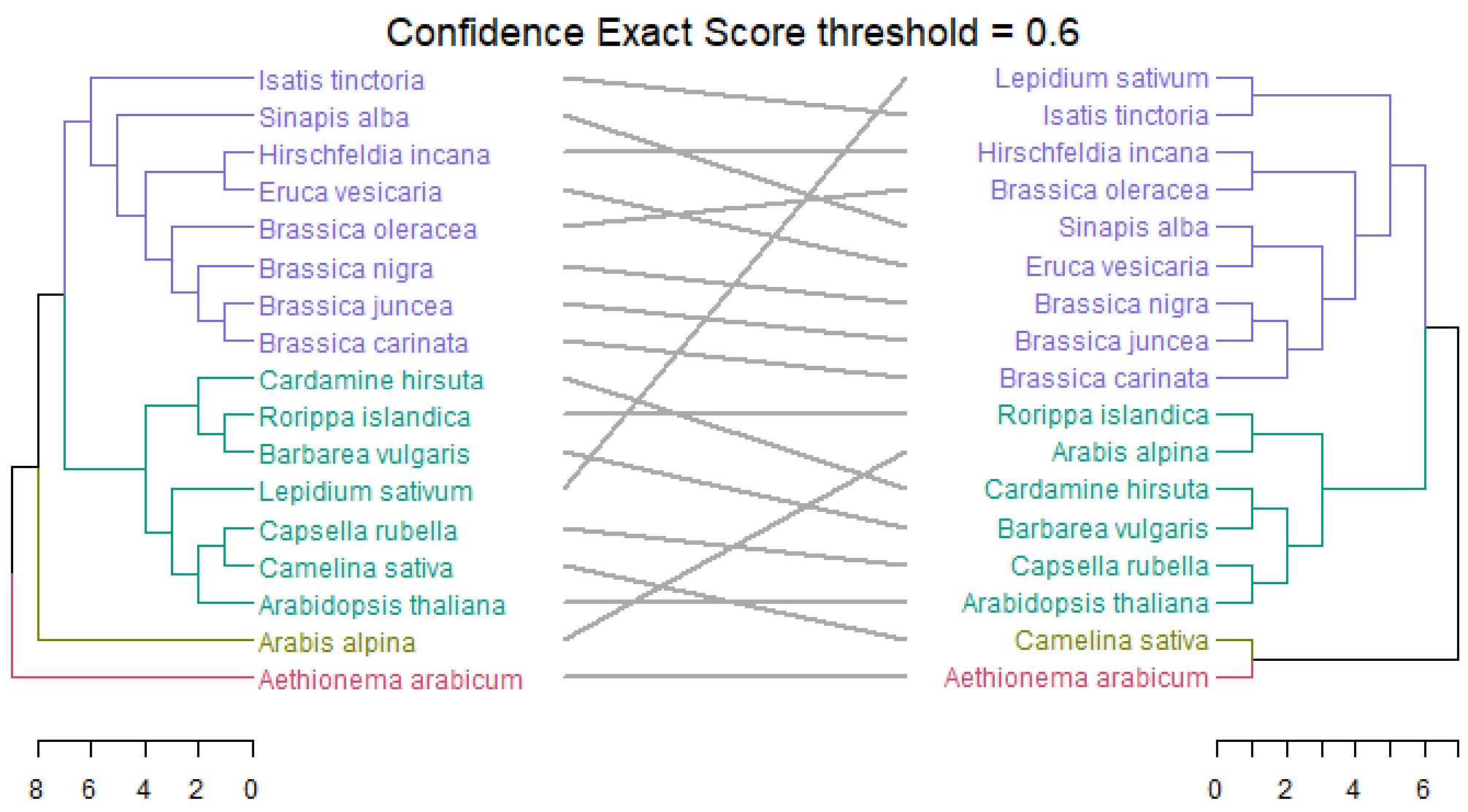
Confidence Exact Score threshold = 0.6
Isatis tinctoria
Sinapis alba
Hirschfeldia incana
Eruca vesicaria
Brassica oleracea
Brassica nigra
Brassica juncea
Brassica carinata
Cardamine hirsuta
Rorippa islandica
Barbarea vulgaris
Lepidium sativum
Capsella rubella
Camelina sativa
Arabidopsis thaliana
Arabis alpina
Aethionema arabicum
Lepidium sativum
Isatis tinctoria
Hirschfeldia incana
Brassica oleracea
Sinapis alba
Eruca vesicaria
Brassica nigra
Brassica juncea
Brassica carinata
Rorippa islandica
Arabis alpina
Cardamine hirsuta
Barbarea vulgaris
Capsella rubella
Arabidopsis thaliana
Camelina sativa
Aethionema arabicum
8 6 4 2 0
0 2 4 6

(n)

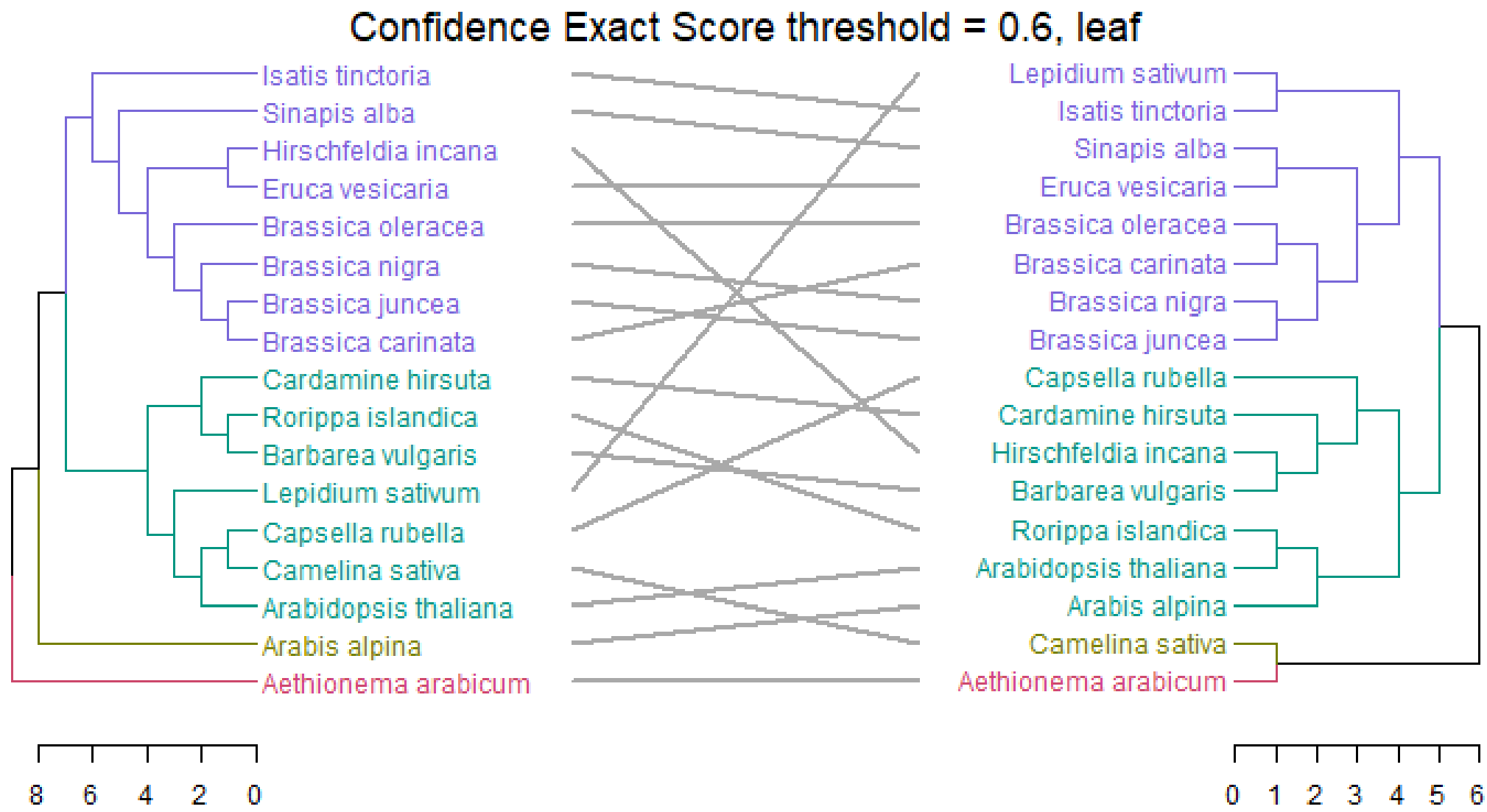

(o)

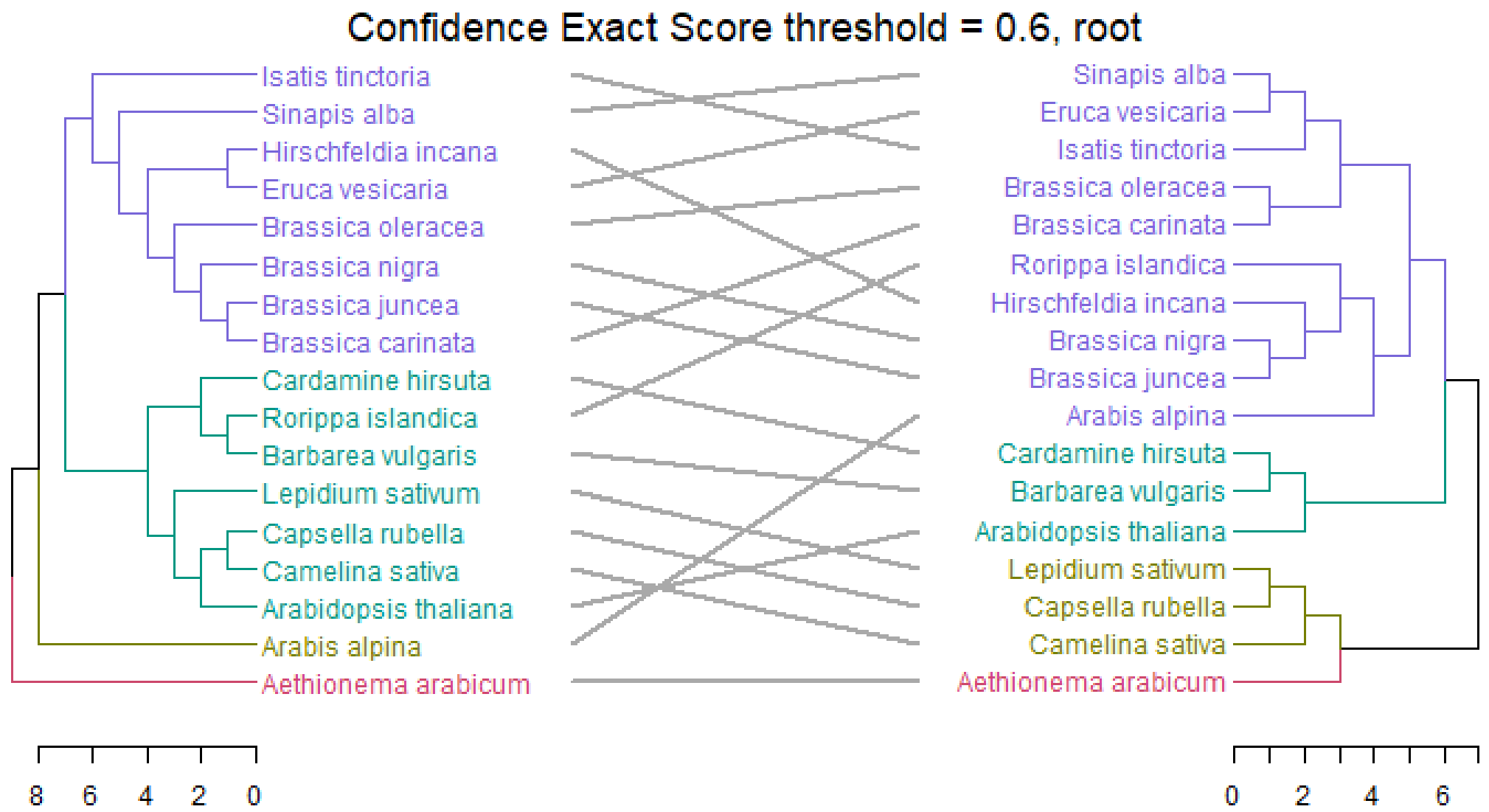

Figure S8: Tanglegrams showing hierarchical clustering of aligned filtered and unfiltered metabolite features across 17 species. **(a)** All detected mass features, unfiltered and aligned with MSCluster in combined positive and negative ionization mode in leaf and root tissue. **(b)** All detected mass features, unfiltered and aligned with MSCluster in combined positive and negative ionization mode in leaf tissue. **(c)** All detected mass features, unfiltered and aligned with MSCluster in combined positive and negative ionization mode in root tissue. **(d)** Detected mass features, aligned with MSCluster in combined positive and negative ionization mode in leaf and root tissue. Features annotated in CANOPUS using the NP.Classifier chemical taxonomy on the "pathway" ontology level were filtered using a probability threshold of 0.6. **(e)** Detected mass features in leaf tissue, aligned with MSCluster in combined positive and negative ionization mode. Features annotated in CANOPUS using the NP.Classifier chemical taxonomy on the "pathway" ontology level were filtered using a probability threshold of 0.6. **(f)**

Detected mass features in root tissue, aligned with MSCluster in combined positive and negative ionization mode. Features annotated in CANOPUS using the NP.Classifier chemical taxonomy on the "pathway" ontology level were filtered using a probability threshold of 0.6. **(g)** Detected mass features, aligned with MSCluster in combined positive and negative ionization mode in leaf and root tissue. Features annotated in CANOPUS using the NP.Classifier chemical taxonomy on the "superclass" ontology level were filtered using a probability threshold of 0.6. **(h)** Detected mass features in leaf tissue, aligned with MSCluster in combined positive and negative ionization mode. Features annotated in CANOPUS using the NP.Classifier chemical taxonomy on the "superclass" ontology level were filtered using a probability threshold of 0.6. **(i)** Detected mass features in root tissue, aligned with MSCluster in combined positive and negative ionization mode. Features annotated in CANOPUS using the NP.Classifier chemical taxonomy on the "superclass" ontology level were filtered using a probability threshold of 0.6. **(j)** Detected mass features, aligned with MSCluster in combined positive and negative ionization mode in leaf and root tissue. Features annotated in CANOPUS using the NP.Classifier chemical taxonomy on the "class" ontology level were filtered using a probability threshold of 0.6. **(k)** Detected mass features in leaf tissue, aligned with MSCluster in combined positive and negative ionization mode. Features annotated in CANOPUS using the NP.Classifier chemical taxonomy on the "class" ontology level were filtered using a probability threshold of 0.6. **(l)** Detected mass features in root tissue, aligned with MSCluster in combined positive and negative ionization mode. Features annotated in CANOPUS using the NP.Classifier chemical taxonomy on the "class" ontology level were filtered using a probability threshold of 0.6. **(m)** Detected mass features in leaf and root tissue, aligned with MSCluster in combined positive and negative ionization mode. Features annotated in SIRIUS v6.1.0 were filtered using a Confidence Exact Score threshold of 0.6. **(n)** Detected mass features in leaf tissue, aligned with MSCluster in combined positive and negative ionization mode. Features annotated in SIRIUS v6.1.0 were filtered using a Confidence Exact Score threshold of 0.6. **(o)** Detected mass features in root tissue, aligned with MSCluster in combined positive and negative ionization mode. Features annotated in SIRIUS v6.1.0 were filtered using a Confidence Exact Score threshold of 0.6.

References

Anaia RA, Chiocchio I, Sontowski R, Swinkels B, Vergara F, van Dam NM. 2025. Ontogeny and Organ-specific Steroidal Glycoside Diversity Is Associated with Differential Expression of Steroidal Glycoside Pathway Genes in Two *Solanum dulcamara* Leaf Chemotypes. *Plant Biology* 27(5):651–68. DOI: https://doi.org/10.1111/plb.13704.

Alberghini B, Zanetti F, Corso M, Boutet S, Lepiniec L, Vecchi A, Monti A. 2022. Camelina [*Camelina sativa* (L.) Crantz] Seeds as a Multi-Purpose Feedstock for Bio-Based Applications. *Industrial Crops and Products* 182:114944. DOI: https://doi.org/10.1016/j.indcrop.2022.114944.

Augustin JM, Brock JR, Augustin MM, Wellinghoff RL, Shipp M, Higashi Y, Kumssa TT, Cahoon EB, Kutchan TM. 2019. Field Performance of Terpene-Producing

Camelina sativa. *Industrial Crops and Products* 136:50–58. DOI: https://doi.org/10.1016/j.indcrop.2019.04.061.

Erthmann PØ, Agerbirk N, Bak S. 2018. A tandem array of UDP-glycosyltransferases from the UGT73C subfamily glycosylate sapogenins, forming a spectrum of mono- and bisdesmosidic saponins. *Plant Molecular Biology* 97(1–2):37–55. DOI: https://doi.org/10.1007/s11103-018-0723-z

Guendouz A, Hannachi A, Benidir M, Fellahi ZEA, Frih B. 2022. Agro-biochemical Characterisation of *Camelina sativa*: A Review. *Agricultural Reviews* 43(3). DOI: http://dx.doi.org/10.18805/ag.RF-230.

Hendriks KP, Kiefer C, Al-Shehbaz IA, Bailey CD, Hooft van Huysduynen A, Nikolov LA, Nauheimer L, Zuntini AR, German DA, Franzke A, Koch MA, Lysak MA, Toro-Núñez Ó, Özüdoğru B, Invernón VR, Walden N, Maurin O, Hay NM, Shushkov P, Mandáková T, Schranz ME, Thulin M, Windham MD, Rešetnik I, Španiel S, Ly E, Pires JC, Harkess A, Neuffer B, Vogt R, Bräuchler C, Rainer H, Janssens SB, Schmull M, Forrest A, Guggisberg A, Zmarzty S, Lepschi BJ, Scarlett N, Stauffer FW, Schönberger I, Heenan P, Baker WJ, Forest F, Mummenhoff K, Lens F. 2023. Global Brassicaceae phylogeny based on filtering of 1,000-gene dataset. *Current Biology* 33(19):4052–68.e6. DOI: https://doi.org/10.1016/j.cub.2023.08.026.

Khakimov B, Tseng LH, Godejohann M, Bak S, Engelsen SB. 2016. Screening for triterpenoid saponins in plants using hyphenated analytical platforms. *Molecules* 21(12):1614. DOI: https://doi.org/10.3390/molecules21121614.

Mach J. 2019. *Camelina*: a history of polyploidy, chromosome shattering, and recovery. *The Plant Cell* 31(11):2550–51. DOI: https://doi.org/10.1105/tpc.19.00754.

Nielsen NJ, Nielsen J, Staerk D. 2010. New resistance-correlated saponins from the insect-resistant crucifer *Barbarea vulgaris*. *Journal of Agricultural and Food Chemistry* 58(9):5509–14. DOI: https://doi.org/10.1021/jf903988f.

Shen J, Günther J, Kjeldgaard-Nintemann S, Cárdenas PD, Bak S. 2025. Metabolic Engineering Reveals LUP5 as a Determinant of Saponin Composition and Insect Resistance in *Barbarea vulgaris*. *bioRxiv*. DOI: https://doi.org/10.1101/2025.08.04.665341v2.

Shinoda T, Nagao T, Nakayama M, Serizawa H, Koshioka M, Okabe H, Kawai A. 2002. Identification of a triterpenoid saponin from a crucifer, *Barbarea vulgaris*, as a feeding deterrent to the diamondback moth, *Plutella xylostella*. *Journal of Chemical Ecology* 28(3):587–99. DOI: https://doi.org/10.1023/A:1014500330510.

Tsugawa H, Nakabayashi R, Mori T, Yamada Y, Takahashi M, Rai A, Sugiyama R, Yamamoto H, Nakaya T, Yamazaki M, Kooke R. 2019. A cheminformatics approach to characterize metabolomes in stable-isotope-labeled organisms. *Nature Methods* 16(4):295–98. DOI: https://doi.org/10.1038/s41592-019-0358-2.

Xu ZY, Xi YF, Zhou WY, Lou LL, Wang XB, Huang XX, Song SJ. 2020. Alkaloids and monoterpenes from the leaves of *Isatis tinctoria* Linnaeus and their chemotaxonomic significance. *Biochemical Systematics and Ecology* 92:104089. DOI: https://doi.org/10.1016/j.bse.2020.104089.

Yuan B, Yang R, Ma Y, Zhou S, Zhang X, Liu Y. 2017. A systematic review of the active saikosaponins and extracts isolated from *Radix Bupleuri* and their applications. *Pharmaceutical Biology* 55(1):620–35. DOI: https://doi.org/10.1080/13880209.2016.1262433.

Zhang L, Parkin IA. 2025. Unveiling unique metabolomic and transcriptomic profiles in three Brassicaceae crops. *Frontiers in Plant Science* 16:1597905. DOI: https://doi.org/10.3389/fpls.2025.1597905.
