# Supplementary material for: Systematic mass-spectrometry-guided metabolic fingerprinting elucidates diversity of specialized metabolites across the Brassicaceae
Source: Metabolomics. 2026 Sep 25;22(5):161. doi: 10.1007/s11306-026-02537-y (PMC13615159; doi:10.1007/s11306-026-02537-y)
Supplement: Supplementary file 2 [file 11306_2026_2537_MOESM2_ESM.docx]

## Supporting Information

### Supplementary material available on Zenodo (DOI: 10.5281/zenodo.19391098)

**Material S1** Instrument settings and mass exclusion list for LC-MS/MS in positive and negative ionization mode.

**Material S2** mzmine 4.8.5 batchfile in XML format, including all settings for feature extraction and alignment, and aligned feature-lists.

**Material S3** MS2LDA analysis. Config file and full results in positive and negative ionization mode.

**Material S4** HTML summaries of computed consensus annotations across SIRIUS v6.1.0, MS2Query v1.0 and Dreams v1.0 against the MassSpecGym library.

**Material S5** Raw and filtered aligned mass features across two batches, including 17 species (leaf and root), using MSCluster. All intermediate files are provided.

**Material S6** Complete annotation table, including all annotation levels (SIRIUS, MS2Query and Dreams against the MassSpecGym library, GNPS library matches, Mass2Motifs, total abundance per sample, taxonomic levels, feature Id) and fully annotated Feature-Based Molecular Network (FBMN)
